# Modulating Enzyme Function *via* Dynamic Allostery within Biliverdin Reductase B

**DOI:** 10.3389/fmolb.2021.691208

**Published:** 2021-05-20

**Authors:** Jasmina S. Redzic, Michael R. Duff, Ashley Blue, Todd M. Pitts, Pratul Agarwal, Elan Zohar Eisenmesser

**Affiliations:** ^1^ Department of Biochemistry and Molecular Genetics, School of Medicine, University of Colorado Denver, Denver, CO, United States; ^2^ Biochemistry and Cellular and Molecular Biology Department, University of Tennessee, Knoxville, TN, United States; ^3^ National High Magnetic Field Laboratory, Tallahassee, FL, United States; ^4^ Division of Medical Oncology, School of Medicine, University of Colorado, Aurora, CO, United States; ^5^ Department of Physiological Sciences and High Performance Computing Center, Oklahoma State University, Stillwater, OK, United States

**Keywords:** enzyme, dynamics, allostery, coupling, reductase, NMR

## Abstract

The biliverdin reductase B (BLVRB) class of enzymes catalyze the NADPH-dependent reduction of multiple flavin substrates and are emerging as critical players in cellular redox regulation. However, the role of dynamics and allostery have not been addressed, prompting studies here that have revealed a position 15 Å away from the active site within human BLVRB (T164) that is inherently dynamic and can be mutated to control global micro-millisecond motions and function. By comparing the inherent dynamics through nuclear magnetic resonance (NMR) relaxation approaches of evolutionarily distinct BLVRB homologues and by applying our previously developed Relaxation And Single Site Multiple Mutations (RASSMM) approach that monitors both the functional and dynamic effects of multiple mutations to the single T164 site, we have discovered that the most dramatic mutagenic effects coincide with evolutionary changes and these modulate coenzyme binding. Thus, evolutionarily changing sites distal to the active site serve as dynamic “dials” to globally modulate motions and function. Despite the distal dynamic and functional coupling modulated by this site, micro-millisecond motions span an order of magnitude in their apparent kinetic rates of motions. Thus, global dynamics within BLVRB are a collection of partially coupled motions tied to catalytic function.

## Introduction

The allosteric regulation of enzyme function is now recognized to occur through multiple mechanisms, which can be mechanical where physical interactions induce long-range conformational changes or dynamic where motions influence networks of partially coupled movements (Motlagh et al., 2014). Methods that are reliant on NMR chemical shifts or evolutionary substitutions have been powerful tools to identify allosteric networks (Selvaratnam et al., 2012a; Selvaratnam et al., 2012b; Gagne et al., 2015; Salinas and Ranganathan, 2018). In contrast, identifying dynamically coupled networks that underly allosteric communication has been more challenging. This is in part due to the fact that dynamics may reflect changes in sampled conformations that are small and are therefore reliant on sensitive experimental methods to directly monitor such changes to what has been called “invisible states” (Kleckner and Foster, 2011). Enzymes are often particularly reliant on dynamics to perform their catalytic functions and dynamically coupled networks that underlie allosteric communication has been identified within several enzymes to date (Doucet, 2011; Duff et al., 2018). Thus, controlling such dynamic allostery may be a powerful way to engineer or fine-tune catalytic function (Doucet, 2011). However, it has also become increasingly recognized that protein dynamics are segmental in nature that lead to partial couplings and often span large distances within proteins (Schlegel et al., 2009; Torbeev et al., 2011; McDonald et al., 2012). Such complicated networks of partially coupled dynamics make it difficult to understand the role of allostery in enzyme function. To address such partially coupled networks, we have previously developed a simple but straightforward strategy that utilizes several criteria for the selection of distally coupled dynamic residues that are then mutated to help understand their roles in allostery and function (Holliday et al., 2017). The first criterion is that these distal residues are inherently dynamic and the second criterion is that they are coupled to active site perturbations, such as exhibiting chemical shift perturbations (CSPs) or dynamic changes upon substrate binding or mutagenesis to the active site. In our initial application of this approach to the isomerase cyclophilin-A, mutations to an inherently dynamic residue 20 Å away from the active site, termed a dynamic “hot spot”, could be mutated in order to control substrate binding in an isomer-specific manner (Holliday et al., 2017). Such “hot spot” mutations induced global dynamic effects that controlled catalytic efficiency and could also be used to map specific networks of dynamically coupled interactions (Holliday et al., 2017). We therefore referred to this approach as Relaxation And Single Site Multiple Mutations (RASSMM) in order to emphasize the fact that we could monitor distal relaxation effects and changes to function by specifically engineering a panel of mutations to one single site. R2-Car-Purcell-Meiboom-Gill (CPMG) dispersion experiments have proven to be a particularly powerful relaxation experiment in monitoring such global changes to dynamics. R2-CPMG dispersion monitors R2 relaxation as a function of an imparted CPMG refocusing field, which provides information regarding rates of motions, populations sampled, and even structural information (kleckner and Foster, 2011; Alderson and Kay, 2020). Here, we have applied this RASSMM approach to biliverdin reductase B (BLVRB) in order to determine whether global motions are allosterically coupled to function within this enzyme and whether enzyme motions may be modulated from a distance to control function in a residue-specific manner.

**FIGURE 1:**
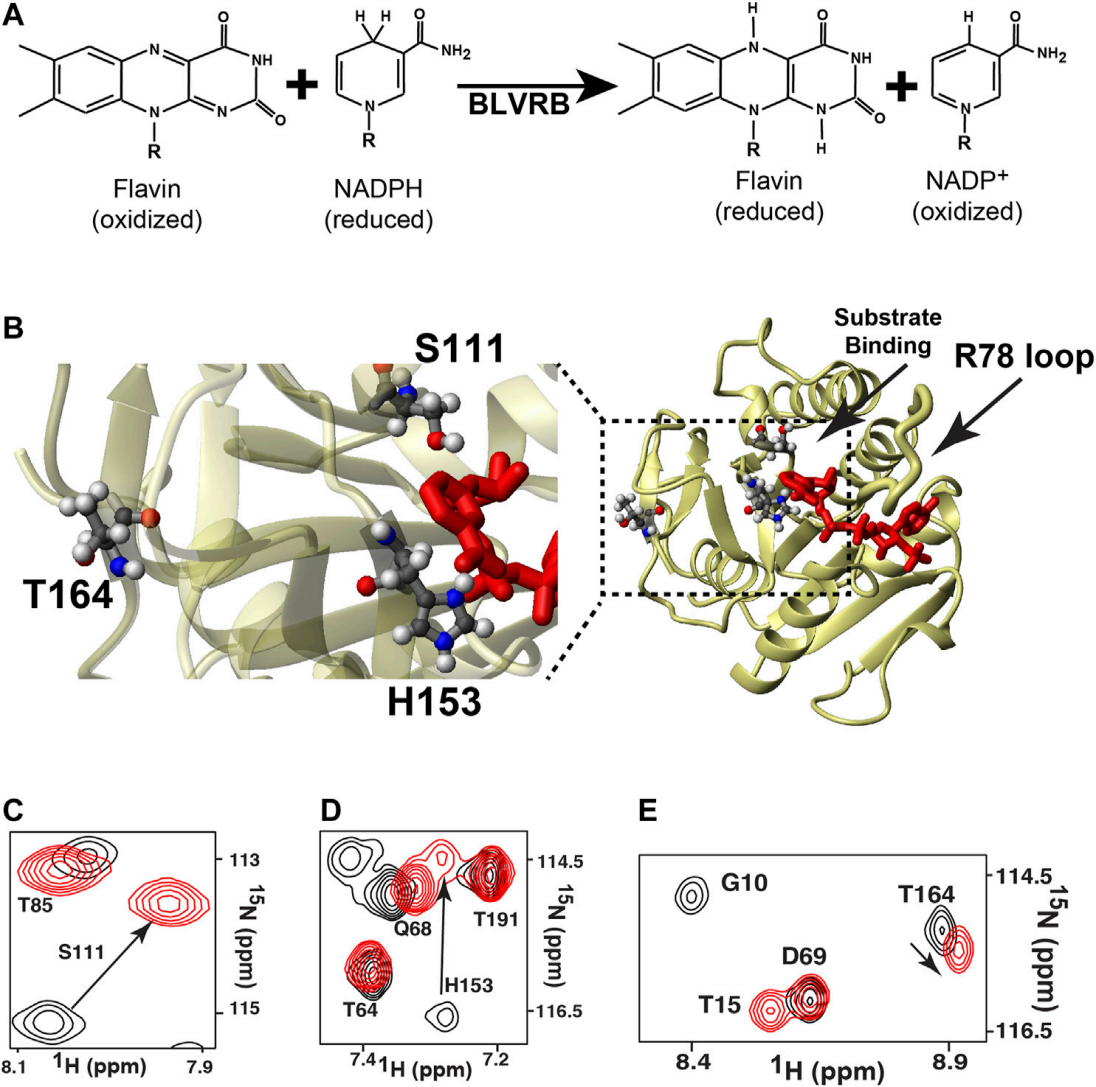
The general reaction mediated by BLVRB and the global impact mediated by coenzyme binding. **(A)** The catalytic reaction of BLVRB comprises the NADPH-dependent reduction of flavins. For flavins, “R” corresponds to different moieties that define the flavin such as FAD or FMN. For NADPH/NADP^+^, only the nicotinamide moiety is shown, and “R” corresponds to the remaining molecule. **(B)** X-ray crystal structure of WT BLVRB, accession number 1HDO (Pereira et al., 2001), along with a blow-up of the active site with residues S111 and H153 and residue T164 that is 15 Å away from the coenzyme. ^15^N-HSQC of apo BLVRB (black) and holo BLVRB (red) of the amides for **(C)** S111, **(D)** H153, and **(E)** T164.

The biliverdin reductase B (BLVRB) family of enzymes are emerging as critical flavin reductases in multiple organisms that range from pathogenic bacteria to humans, as their flavin substrates act as redox sensors and coenzymes for many other enzymes (Vervoort and Rietjens, 1996; McDonagh, 2001; Becker et al., 2011; Huijbers et al., 2014; Adak and Begley, 2017). The BLVRB enzyme family catalyze the NADPH-reduction of bilirubin but also multiple flavins (Figure 1A), which includes flavin adenine dinucleotide (FAD) and flavin mononucleotide (FMN). In humans, redox regulation *via* BLVRB (NCBI Reference Sequence: NP_000704.1) is so important that this enzyme alone can dictate hematopoietic cell fate (Wu et al., 2016). This critical role is also consistent with our discovery of high levels of BLVRB expression in red blood cells (Paukovich et al., 2018). Mechanistically, we have previously discovered that coenzyme binding is orders of magnitude tighter than substrate binding (Paukovich et al., 2018), which is in contrast to the well-known dihydrofolate reductase (DHFR) where both coenzyme and substrate bind relatively tightly (Fierke et al., 1987). However, both BLVRB and DHFR share several common catalytic features. For example, both enzymes share similar mechanisms where bulk water is used for an initial protonation step of the substrate followed by hydride transfer from the coenzyme to the substrate (Smith et al., 2008; Liu et al., 2014). Another similarity is that despite their completely different tertiary structures, both DHFR and BLVRB rely on loop closure for function. Specifically, the “M20 loop” of DHFR dynamically modulates its activity (Boehr et al., 2006; Bhabha et al., 2013; Singh et al., 2015; Hughes et al., 2017) and we have recently shown that the “R78 loop” of BLVRB dynamically modulates its activity (Paukovich et al., 2018; Figure 1B). Thus, loop closure within these reductases provides a unique environment for their catalytic transfer of a hydride from one molecule (the coenzyme) to another (the substrate) and their activities are not necessarily reliant on a single residue for catalysis. This is likely why attempts to identify a “catalytic residue” within BLVRB have been unsuccessful, which include BLVRB active site mutants of S111 and H153 that result in diminished function but not functional knockouts (Smith et al., 2008; Chu et al., 2017). DHFR is similar in that catalysis is a collective of multiple residues that contribute to the active site environment, as elucidated by elegant studies that have shown residues such as DHFR D27 and Y100 exhibit synergistic effects (Liu et al., 2014). However, while distal dynamic residues within DHFR modulate active site motions and function (Watt et al., 2007; Mauldin et al., 2012; Duff et al., 2018), such global coupling remains unknown for BLVRB.

To determine whether distally coupled dynamic residues modulate active site motions and function in BLVRBs, here we used the RASSMM approach on human BLVRB along with comparative relaxation studies of lemur BLVRB (NCBI Reference Sequence: XP_020138941.1). Our previous studies had suggested that a distal site, T164, is both inherently dynamic and coupled to the active site, which would fulfill the requirements for selecting a residue to then mutate in accord with the RASSMM approach. For example, coenzyme binding induces chemical shift perturbations (CSPs) to active site residues such as S111 and H153, but also CSPs to distal sites that included T164 within the C-terminal lobe (Figure 1C). Here, both ensemble calculations and active sites mutants corroborated the inherent dynamics and active site coupling of this T164 site, which are the requirements for the selection of residues to select for the RASSMM approach. Considering that the second step in the RASSMM approach requires mutating this identified site to a series of residues, we used evolution as a guide to help select site-specific mutations. Specifically, we discovered that position 164 within mammalian BLVRBs toggles between a threonine and serine and that dynamics are completely quenched within the lemur BLVRB homologue that comprises only one single substitution within this region, which is S164. Thus, in accord with the second step of the RASSMM approach, multiple mutations to human BLVRB T164 that included a serine were engineered and further corroborated coupling to the active site through both CSPs and R2-CPMG dispersions. Such allosteric changes that could be altered by mutations to T164 were found to increase coenzyme affinity. We have therefore discovered that this distal site may be modulated by either evolution or mutagenesis to allosterically control function.

## Results

### Ensemble Calculations Identify the Conformational Plasticity Measured *via* NMR Relaxation

**FIGURE 2:**
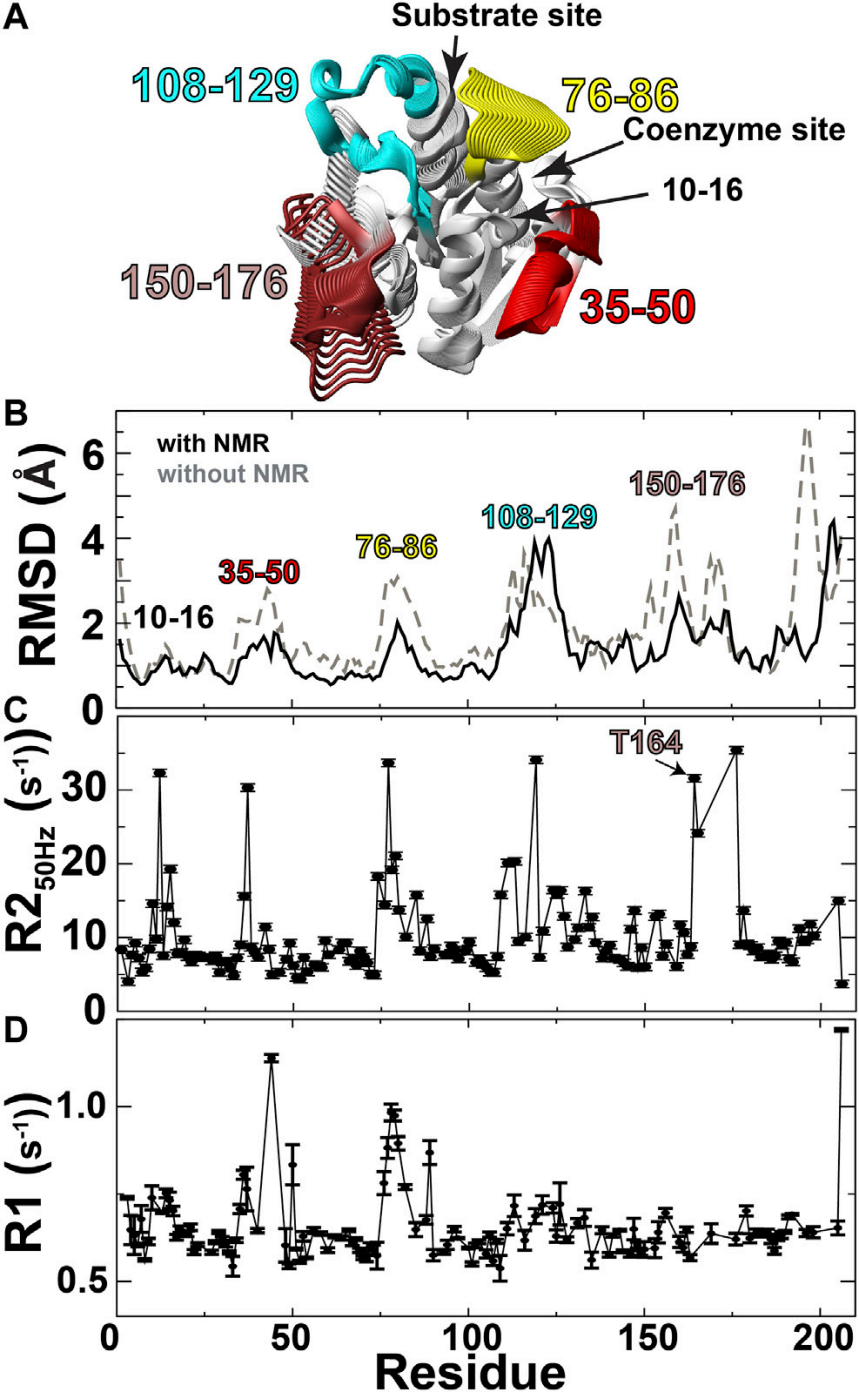
Solution ensembles identify structural heterogeneity. **(A)** Structural ensembles were used to calculate a continuum of potentially sampled conformations using MovieMaker (Maiti et al., 2005). **(B)** Backbone RMSDs calculated both with (black line) and without (grey-dashed line) NMR data. **(C)** R2 relaxation rates extracted from the first 50 Hz refocusing time point of R2-CPMG relaxation dispersion data. **(D)** R1 relaxation rates of apo BLVRB.

We first sought an atomic-resolution description of BLVRB dynamics through structural studies in order to identify distal residues to the active site that are inherently dynamic, which is the first criterion of the RASSMM approach. While previous relaxation studies indicate that several regions within BLVRB exhibit chemical exchange (Paukovich et al., 2019), the underlying physical exchange process that induces chemical exchange may be due to dynamics of these regions themselves or neighboring regions. Structural studies are particularly challenging with inherently dynamic proteins such as BLVRB, as high-resolution structural data are difficult to obtain. For example, the difficulty in crystallizing apo BLVRB has been ascribed to its dynamic nature (Chu et al., 2017), which is consistent with our previous studies that have identified millisecond-microsecond (μs-ms) timescale exchange monitored through R2-CPMG relaxation dispersion largely quenched within the holo enzyme (Paukovich et al., 2018). In fact, the resonances from several regions such as residues 167–175 and 199–204 are simply not observed, potentially owing to exchange on an intermediate timescale (Paukovich et al., 2018). Thus, as an alternative structural approach, chemical shift-based methods in conjunction with sparse NOEs were used here to guide solution ensemble calculations, which have been shown to produce structures with remarkable accuracy and precision as we and others have shown (Rosato et al., 2012; Kendrick et al., 2014; Holliday et al., 2017). Specifically, we used CS-Rosetta that utilizes fragment libraries to build structural ensembles and has been successfully used for proteins almost twice as large as BLVRB (Lange et al., 2012). While such calculations may therefore be biased toward known structures, they represent plausible conformations consistent with structural data (chemical shifts, NOEs), which can be directly compared to relaxation data that monitors motions on multiple timescales. To obtain these low-resolution structural ensembles (Figure 2; Table 1), we employed several experimental restraints described in Materials and Methods, which included sparse NOEs (Supplementary Table S1).

**TABLE 1:** Structural statistics for the apo BLVRB CS-rosetta structures. NOE restraints were exported from CCPNmr as exact distances used for calculations. The conserved SDR domain comprises residues 1–150.

No. of residues	206
Chemical Shift restraints	1,338
N/HN	83/182
CA/HA	193/175
CB/HB	174/177
NOE distance restraints	143
Avg violation per structure	23 ± 3
Avg violation distance (Å)	0.27 ± 0.01
Medium-range (|i−j| < 4)	73
Long-range (|i−j| > 5)	70
Average rmsd	
Backbone, 1–206 (Å)	2.58 ± 0.68
Backbone, 1–150 (Å)	2.00 ± 0.68
Ramachandran plot summary (%)	
Most favored regions	83.4
Allowed regions	16.6

Ensemble calculations of apo BLVRB identify potential dynamic regions that predominantly include the active site along with residues 156–176 within the C-terminal lobe that comprises residues 150–206 (Figure 2). Specifically, CS-Rosetta was used to calculate solution ensembles of apo BLVRB using NMR chemical shifts (BMRB accession 27,462) and supplemented with sparse NOEs (Supplementary Table S1). These ensemble calculations are shown here as a continuum (Figure 2A), which pictorially highlights the dynamic regions that include the active site and residues 156–176 within the C-terminal lobe. Ensemble calculations using experimental restraints result in better precision (i.e., smaller RMSDs) than those calculated in the absence of any experimental NMR data (Figure 2B, black line vs. grey-dashed line, respectively), indicating that the experimental data helps define the solution ensembles. The RMSD of these experimentally driven structural ensembles was then compared to NMR relaxation data. These comparisons include both μs-ms motions monitored *via* R2 relaxation (Figure 2C) and ps-ns motions monitored *via* R1 relaxation, as previously measured (Paukovich et al., 2018; Figure 2D). Conformationally heterogeneous regions within the calculated ensembles were largely corroborated by these NMR relaxation measures, as they were confirmed to be mobile in either the faster ps-ns timescale (residues 35–50 and 76–86) and/or slower μs-ms timescale (residues 76–86, 108–129, and 156–176). Residues 10–16 likely exhibit chemical exchange due to the R78 loop that samples both open/closed conformations within the solution ensembles. Finally, Thus, in accord with the RASSMM approach that first seeks to identify distally dynamic regions to the active site, both our previous relaxation experiments and these data-driven ensembles indicate that residues within this region of 156–176 satisfy this first criterion.

### Active Site Mutants are Globally Coupled

**FIGURE 3:**
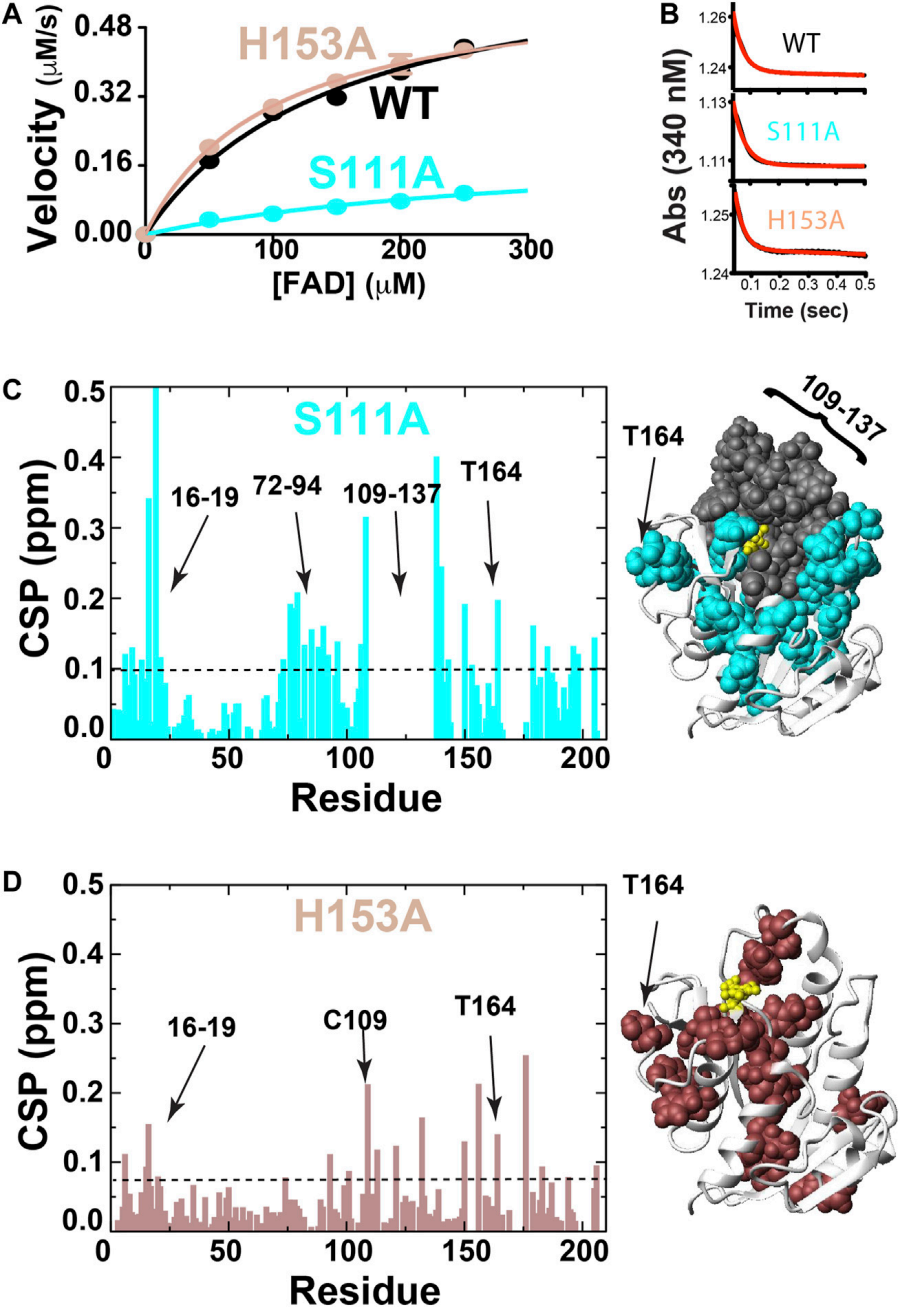
BLVRB active site mutations S111A and H153A. **(A)** Michaelis-Menten kinetics monitored for BLVRB WT, S111A, and H153A. **(B)** Pre-steady-state kinetics for BLVRB WT, S111A, and H153A. **(C)** CSPs between apo BLVRB WT and apo BLVRB S111A with the line indicating 0.1 ppm that is the average plus ½ of the standard deviation. Residues for which their corresponding resonances were not found are also shown (gray). **(D)** CSPs between apo BLVRB WT and apo BLVRB H153A with the line indicating 0.075 ppm that is the average plus ½ of the standard deviation.

In order to address the second criterion of the RASSMM approach that seeks to identify inherently dynamic regions coupled to the active site, we sought to mutate specific residues to monitor their distal effects. Already, we knew that coenzyme binding induces changes to distal sites, yet the conformational rearrangements are quite extensive that provided the impetus here to make site-specific changes. We selected BLVRB S111A and H153A, which have previously been shown to alter BLVRB function through kinetics studies that indicate the underlying reasons are due to diminished substrate affinity for S111A and diminished coenzyme affinity for H153A (Chu et al., 2017; Smith et al., 2008). We confirmed that both mutants are active in reducing FAD, which resulted in the expected reduction to substrate affinity solely for the S111A mutation (Figure 3A; Table 2). Interestingly, neither mutation significantly alters the chemical step of hydride transfer (k_hyd_, Figure 3B and Table 2), which was assessed through pre-steady-state kinetics as originally described by Farnum et al. (1991) and more recently for our studies with BLVRB (Duff et al., 2020). Thus, while H153A and S111A mutations have been shown to modulate function through variations in coenzyme or substrate binding affinities, respectively (Smith et al., 2008; Chu et al., 2017), we show here that they do not affect the chemical step. The reason that we emphasize this is that our previous application of the RASSMM approach identified a distally dynamic site within the proline isomerase cyclophilin-A that also altered modulated substrate affinity but did not appear to modulate the chemical step (Holliday et al., 2017).

**TABLE 2:** Individual catalytic parameters for BLVRB mutations.

Human BLVRB	k_hyr_ ^a^ (s^−1^)	K_M_ ^b^ for FAD (μM)	k_cat_ ^c^ (s^−1^)
WT^c^	28 ± 5	151 ± 18	0.068 ± 0.004
S111A	33 ± 8	325 ± 96	0.021 ± 0.004
H153A	30 ± 7	98 ± 11	0.059 ± 0.003
T164A	36 ± 7	153 ± 18	0.063 ± 0.002
T164I	33 ± 12	156 ± 18	0.049 ± 0.003
T164S	34 ± 5	127 ± 15	0.039 ± 0.003

a Hydride transfer rates were determined from pre-steady-state UV-kinetics.

b Michaelis-Menten constants and turnover-rates were determined from steady-state UV-kinetics.

c Initially reported in Duff et al. (2020).

Both BLVRB mutations S111A and H153A impart large-scale changes, as monitored through CSPs relative to the WT BLVRB (Figures 3C,D). Interestingly, much of the active site that we have previously shown undergoes conformational exchange in human wild type (WT) BLVRB, exhibits a complete loss in signal within the S111A mutation that includes residues 109–137 (Figure 3C). Thus, while we have previously shown that much of the active site of WT BLVRB exhibits an inherent chemical exchange on the fast timescale through R2-CPMG relaxation dispersion (Paukovich et al., 2018), S111A shifts the timescale of this regime from a fast to an intermediate exchange. This means that S111A is globally coupled through dynamics in addition to structural perturbations monitored through CSPs. The H153A mutation also imparts distal changes (Figure 3D). Although the CSPs induced by H153A are smaller than those induced by S111A, many of these distally coupled residues are similar. Specifically, the resonance of T164 is perturbed in the context of both mutations (Figures 3C,D), indicating that these active site residues are both coupled to T164. Thus, in addition to its inherent dynamics, T164 further fulfills the criteria of the RASSMM approach in that it is also coupled to the active site.

### Dynamic Differences between Human and Lemur BLVRBs Include Position 164

**FIGURE 4:**
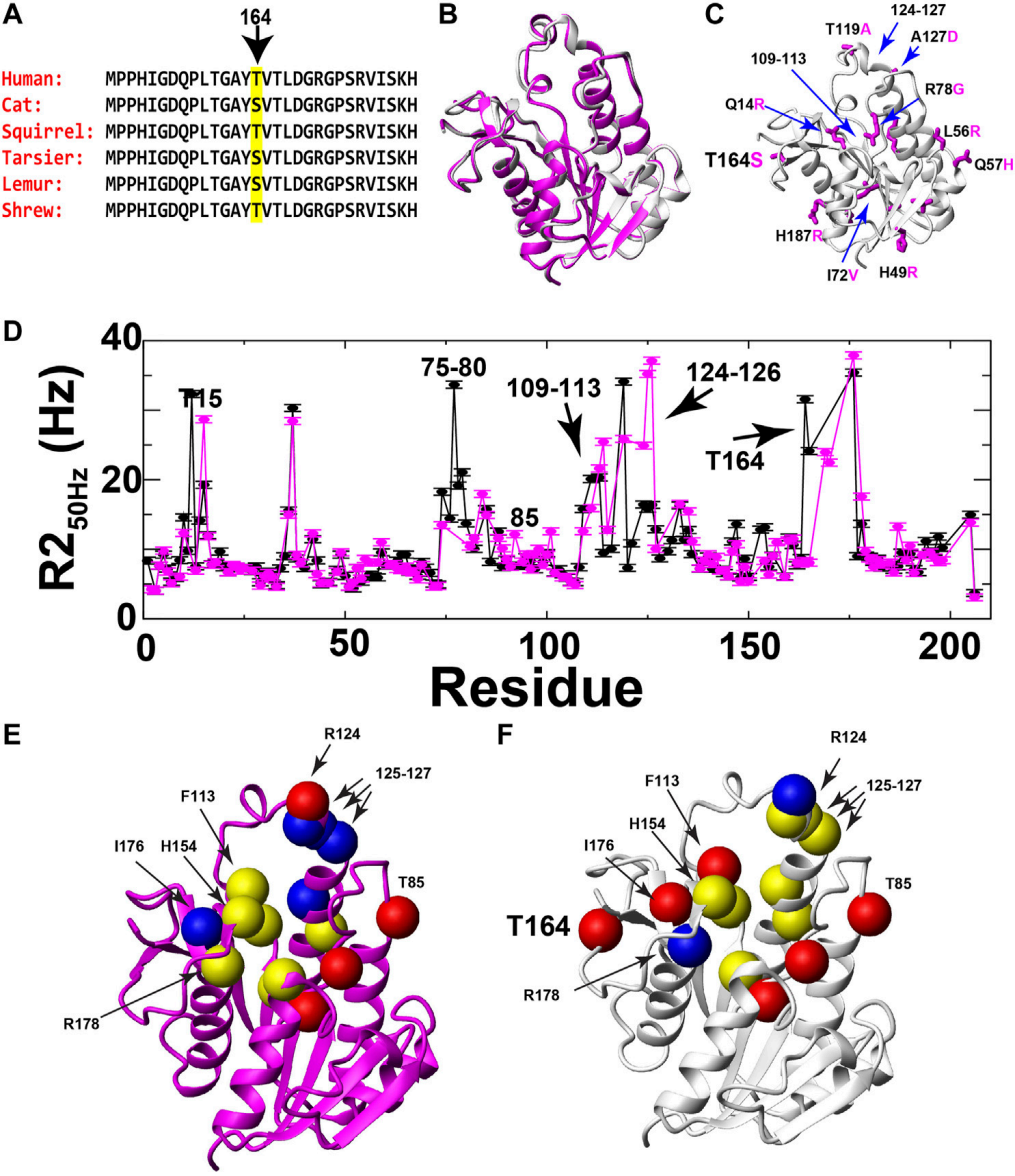
Evolutionary changes of BLVRB position 164 and comparative dynamics of human and lemur BLVRB family members. **(A)** Sequence comparison of six mammalian BLVRBs for residues 151–169 shown from top (most recent) to bottom as a function of their most recent evolutionary branch point as described by Hallstrom and Janke (2010). Position 164 is highlighted (yellow). **(B)** Superposition of human and lemur BLVRBs (PDB accession numbers 1HDO and 6OQG, respectively). **(C)** Lemur BLVRB comprises 16 amino acid changes mapped onto human BLVRB (magenta) with several of these amino acids highlighted that include position 164 (human in black, lemur in magenta). **(D)** R2 relaxation rate at 50 Hz CPMG refocusing for human (black) and lemur (magenta) as a function of residue. **(E)** Individually extracted rates of exchange (kex) for lemur BLVRB amides are shown as balls for rates less than 1000 s^−1^ (blue), from 1000 to 3000 s^−1^ (yellow), and higher than 3000 s^−1^ (red). **(F)** Individually extracted rates of exchange (kex) for the same residues for human BLVRB amides are shown as balls for rates less than 1000 s^−1^ (blue), from 1000 to 3000 s^−1^ (yellow), and higher than 3000 s^−1^ (red). Only residue human T15 is not shown as the exchange contribution was too low to fit (Supplementary Figure S1).

While identifying human BLVRB T164 as an ideal site to mutate for the RASSMM approach, we also noticed that this position is evolutionarily dynamic and realized that evolution may help serve as a guide for the specific selection of residues to mutate. Specifically, a sampling of mammalian sequences at sequential branch points according to Hallstrom and Janke (2010) reveals that position 164 toggles between a threonine and serine (Figure 4A). Thus, prior to specifically mutating human T164 in order to address its global changes through the RASSMM approach, we first sought evolutionary insight by comparing the dynamics between homologues that differ in this position. We used lemur BLVRB that comprises a serine at position 164 (S164) instead of a threonine as its human counterpart (T164). Our previous studies revealed that the lemur BLVRB exhibits similar fast timescale dynamics to human BLVRB identified *via* R1 relaxation and a nearly identical holo enzyme structure (Figure 4B; Duff et al., 2020). However, we have previously discovered that a single mutation to cyclophilin-A can induce global dynamic changes to slower μs-ms timescale motions that modulate function (Doshi et al., 2016; Holliday et al., 2017). As lemur BLVRB comprises 16 substitutions within its 206 residues relative to human BLVRB that includes position 164, which is inherently dynamic and coupled to the active site within human BLVRB (Figure 4C), we sought here to compare the μs-ms dynamics between the two homologues.

Despite similar fast timescale motions and X-ray crystal structures, R2 relaxation measured here reveals that human and lemur BLVRBs differ in their inherent μs-ms timescale motions. Specifically, R2-CPMG dispersions were collected for lemur BLVRB and compared to human BLVRB (Supplementary Figure S1). Overall differences are illustrated by comparing R2 relaxation rates from the lowest R2-CPMG refocusing field of 50 Hz that comprises the highest contribution from exchange (Figure 4D). One of the most dramatic differences to the inherent motions include a near complete quenching of R2s within lemur BLVRB S164 relative to human BLVRB T164 that is discussed further below. The localized nature of motions can also be observed within the R2-CPMG dispersion profiles themselves that are a mixture between fast movements that give rise to linear dispersions and slower motions that give rise to sigmoidal profiles (Supplementary Figure S1). Even neighboring residues often exhibit a complicated mixture of differential dynamics that suggests caution in accurately extracting the explicit exchange rates when such nuclei are likely sensing multiple chemical exchange phenomena. However, single nuclei were fit in order to provide an estimate of their exchange rates that are mapped onto their respective the X-ray crystal structures of their holo forms (Figures 4E,F). For example, several exchange phenomena of neighboring residues within the active site exhibit similar motions in both BLVRB homologues, such as C109/S111, I133/M135, and residues 125–127 that result in similar kinetic rates for both homologues of ∼1500 s^−1^ (Supplementary Figure S1). Despite the similar exchange for residues 125–127 that comprise the substrate binding site, residues 124–126 appear to have two exchange phenomena in lemur BLVRB. Specifically, residues 124–126 have an R2-CPMG field dependence that indicate μs-ms timescale motions but retain a high R2 at the highest imparted CPMG field that indicates an additional faster exchange process within the μs timescale (Supplementary Figure S1). These findings alone are meaningful, as they reveal a shift to an additional exchange phenomenon within lemur BLVRB relative to that of human BLVRB. The selection of residues chosen to globally fit are described in later sections upon applying the RASSMM approach to site 164 that identifies potentially coupled motions. However, it is important to point out here that similar fast timescale motions with similar structures across homologues but with very different μs-ms timescale motions have been reported for the cyclophilin-A family just as we report here for BLVRB family members (Holliday et al., 2015a).

Potentially the most striking change in R2 relaxation identified here is that measured at position 164 where exchange is observed within human BLVRB T164 yet is completely quenched in the context of lemur BLVRB S164 (Figures 4E,F; Supplementary Figure S1). There are no evolutionary substitutions within close proximity to position 164 between these two homologues (Figure 4C), suggesting that this is a true change to the inherent dynamics of this region caused by its substitution.

### Evolutionarily Guided Mutations to Human BLVRB T164 Incur the Largest Functional Impact and Suggests That Coenzyme Binding is Coupled to the Rate-Limiting Step

**FIGURE 5:**
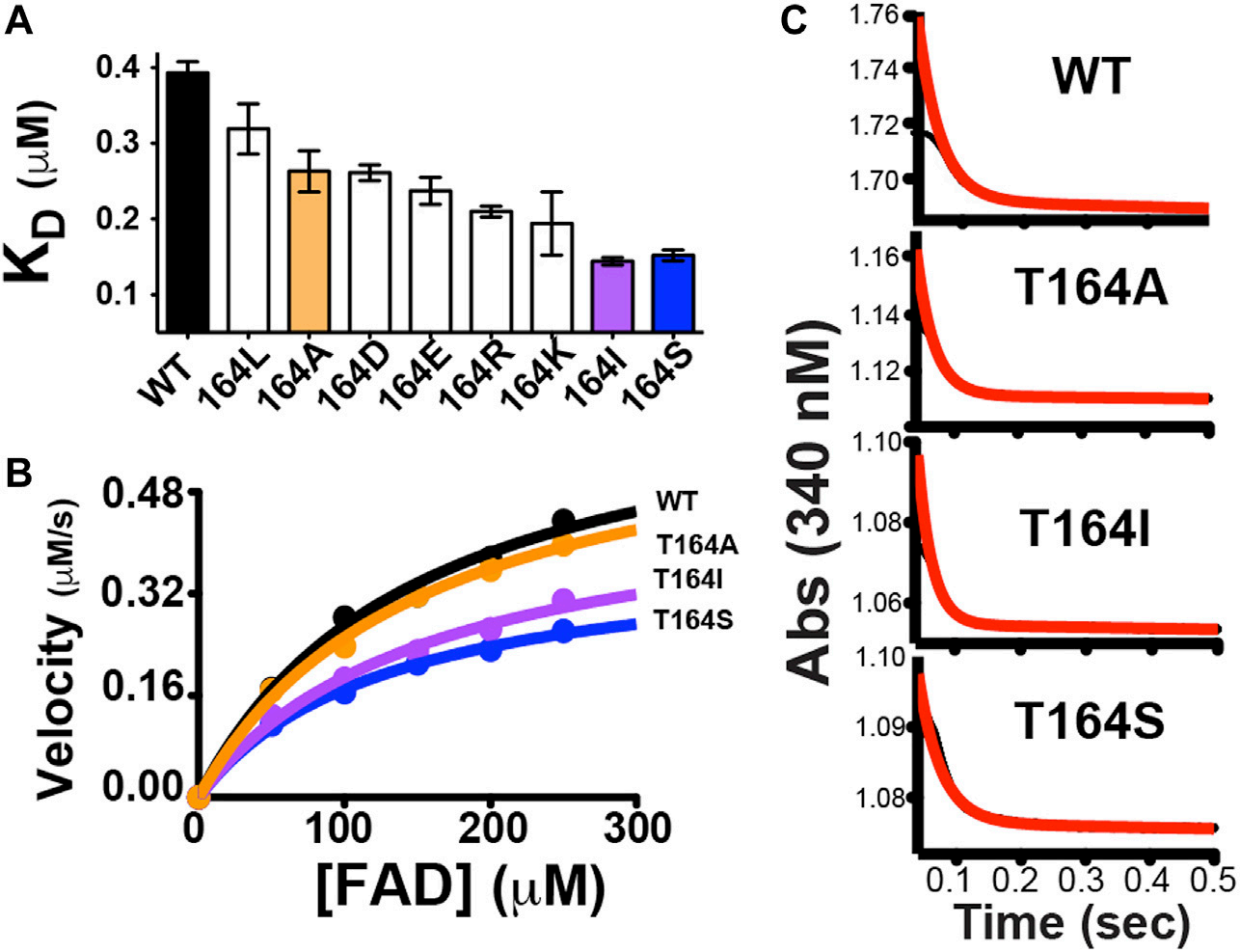
Functional changes induced by point mutations to human BLVRB T164. **(A)** A panel of mutations was screened for their dissociation constants (K_D_) to NADP^+^ using ITC, as previously described. Mutations that were selected for further analysis are colored for WT BLVRB (black), T164A (orange), T164I (purple), and T164S (blue). **(B)** Steady-state kinetics for BLVRB WT (black), T164A (orange), T164I (purple), and T164S (blue). **(C)** Pre-steady-state kinetics BLVRB WT, T164A, T164I, and T164S at the lowest FAD concentration used.

As the first step of the RASSMM approach identified human BLVRB T164 as both inherently dynamic and coupled to the active site, we next sought to address the second step of the RASSMM approach that imparts multiple mutations to this single site. While we engineered a panel of mutations to this site that include hydrophobic residues and hydrophilic residues, evolutionarily guided mutations were also considered based on our findings above with lemur BLVRB. Specifically, the conservative mutation of T164→S was also engineered based on the evolutionary comparisons within mammalian BLVRBs. We first probed the panel of BLVRB single-site mutants for their role in coenzyme binding using isothermal titration calorimetry (ITC). Surprisingly, one of the largest imparted changes to coenzyme affinity that incurred an approximately 2-fold increase was for that of the conservative mutation of T164S (Figure 5A). The other mutation with a similar functional impact on coenzyme binding is T164I. Interestingly, an isoleucine is found within multiple insect species that include *Aedes aegypti* BLVRB (NCBI Reference Sequence: XP_001649677.1), although insect BLVRBs are dramatically different from their mammalian counterparts in primary sequence that make it more difficult to pinpoint the role of specific evolutionary changes (<50% sequence identity). Nonetheless, these binding studies immediately identify an allosteric role for position 164 within human BLVRB with the most dramatic changes to coenzyme affinity also being identical to evolutionary changes.

In order to further examine the functional role of position 164 and simultaneously use these allosteric changes to potentially help address the rate-limiting step of BLVRB catalysis, we selected a subset of mutations that represent a minimum change in coenzyme affinity (T164A) and a larger change in coenzyme affinity (T164I, T164S). Because the coenzyme binds to BLVRB several orders of magnitude tighter than the flavin substrates, the rate-limiting step has been presumed to be coenzyme binding (Paukovich et al., 2018). This is supported here by mutations at the T164 site using steady-state kinetics of substrate turnover (Figure 5B). Specifically, substrate turnover was similar between WT BLVRB and the T164A mutant, yet turnover was significantly diminished for BLVRB, T164I, and T164S. Considering that the substrate K_M_ values were similar for all T164 mutations (Table 2), this suggests that the tighter binding of the coenzyme (and likely a slower off-rate) is rate-limiting for substrate turnover. Mutations to T164 do not measurably alter the chemical step of hydride transfer, as monitored through pre-steady-state kinetics (Figure 5C), thereby further narrowing the role of allosteric coupling through T164 to the changes monitored in its coenzyme interactions. Thus, analogous to our first application of RASSMM where distal mutations were coupled to substrate binding (Holliday et al., 2017), a similar phenomenon is identified here where distal mutations are coupled to coenzyme binding in BLVRB.

### Mutations to Human BLVRB T164 Modulate Global Dynamics

**FIGURE 6:**
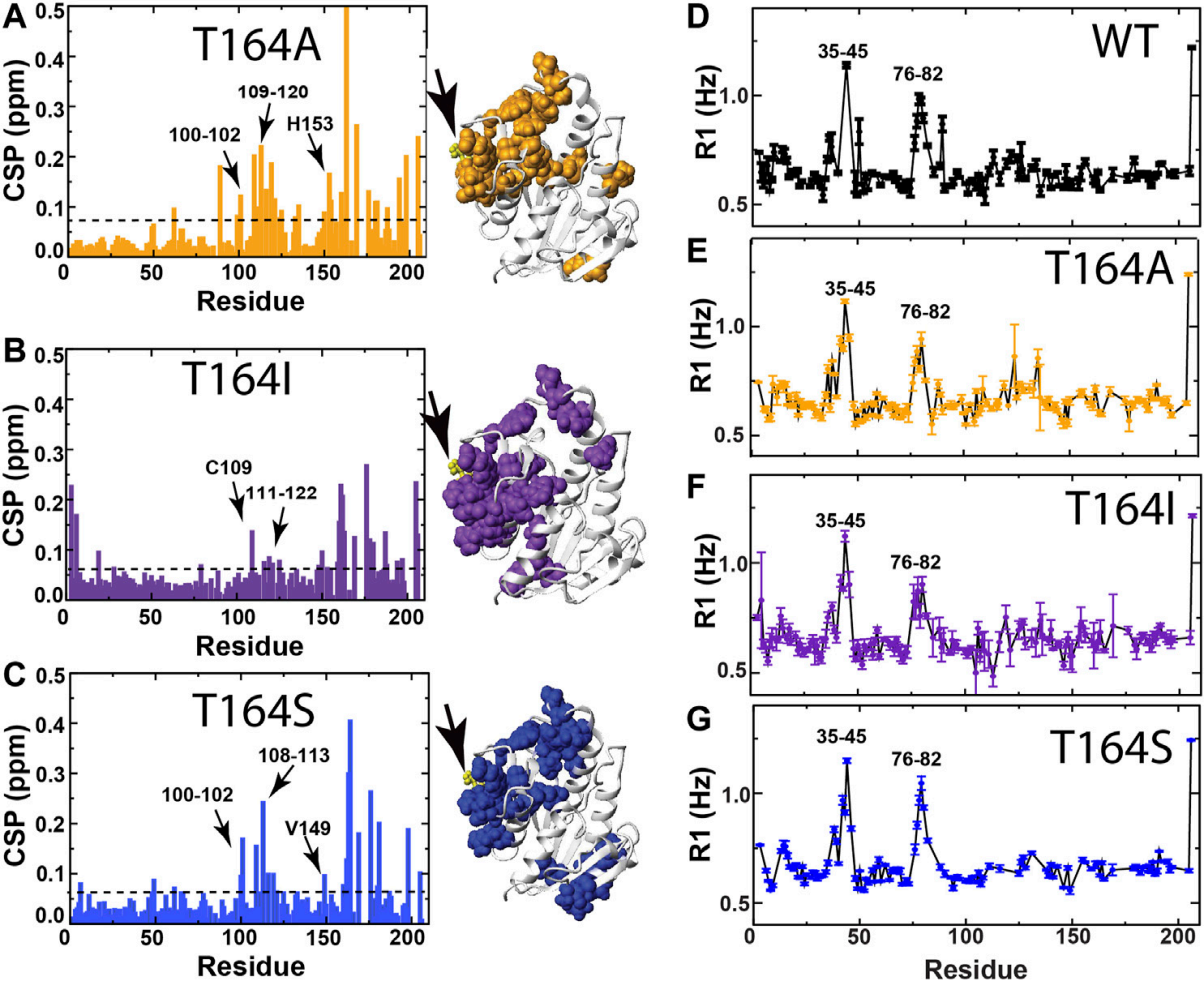
CSPs and R1 relaxation rates of BLVRB T164 mutants. **(A)** CSPs between apo BLVRB WT and apo BLVRB T164A with the line indicating 0.08 ppm that is the average plus ½ of the standard deviation. **(B)** CSPs between apo BLVRB WT and apo BLVRB T164I with the line indicating 0.07 ppm that is the average plus ½ of the standard deviation. **(C)** CSPs between apo BLVRB WT and apo BLVRB T164S with the line indicating 0.07 ppm that is the average plus ½ of the standard deviation. **(D)** R1 relaxation rates for BLVRB WT previously published (Paukovich et al., 2018). **(E)** R1 relaxation rates for BLVRB T164A. **(F)** R1 relaxation rates for BLVRB T164I. **(G)** R1 relaxation rates for BLVRB T164S. All CSPs above the line are mapped onto the X-ray crystal structure (PDB accession 1HDO) and all data were collected at 900 MHz at 20°C.

At the heart of the RASSMM approach is the potential for identifying similar relaxation effects to multiple residues imparted by variations at the single mutation site (i.e., networks), such as those imparted by mutations to human BLVRB T164. Thus, we once again chose to further probe human BLVRB T164A (minimum functional change) and both BLVRB, T164I ,and T164S (maximum functional changes). CSPs can also provide a means to identify coupled networks, yet many of the perturbed residues are similar for all three mutations with variations primarily to their magnitudes (Figures 6A–C). Furthermore, CSPs induced by T164 mutations do not appear to induce similar changes to that of coenzyme binding. For example, we employed an NMR chemical shift projection analysis (CHESPA) that has been successfully used to monitor global changes that reflect conformational shifts to sampling of active states, which includes changes induced by mutations (Selvaratnam et al., 2012a; Selvaratnam et al., 2012b; Gagne et al., 2015). Specifically, by comparing CSPs induced upon human WT BLVRB binding to the coenzyme to CSPs induced by the T164 mutations (Supplementary Table S2), only a subset of residues largely localized near position 164 within the C-terminal lobe exhibited any covariance at all (Supplementary Table S3). Interestingly, there is a negative correlation for several residues within the T164A active site (Supplementary Table S3, residues 119–120 and H153). This could theoretically suggest that dynamic changes imparted by T164 mutations for T164S and T164I that increase coenzyme affinity may be negated by structural changes within T164A. However, overall there is no clear direct relationship between function induced by T164 mutations and CSPs. Additionally, R1 relaxation rates are largely similar for these three mutations that indicate similar ps-ns motions as WT BLVRB (Figures 6D–G). For example, locally elevated R1 relaxation rates for residues 35–45 and 76–82 are consistent to that previously reported for human and lemur BLVRBs (Paukovich et al., 2018; Duff et al., 2020 site). Thus, we next focused on μs-ms timescale motions using R2-CPMG dispersion that has previously proven successful in identifying coupled networks within cyclophilin-A (Holliday et al., 2017).

**FIGURE 7:**
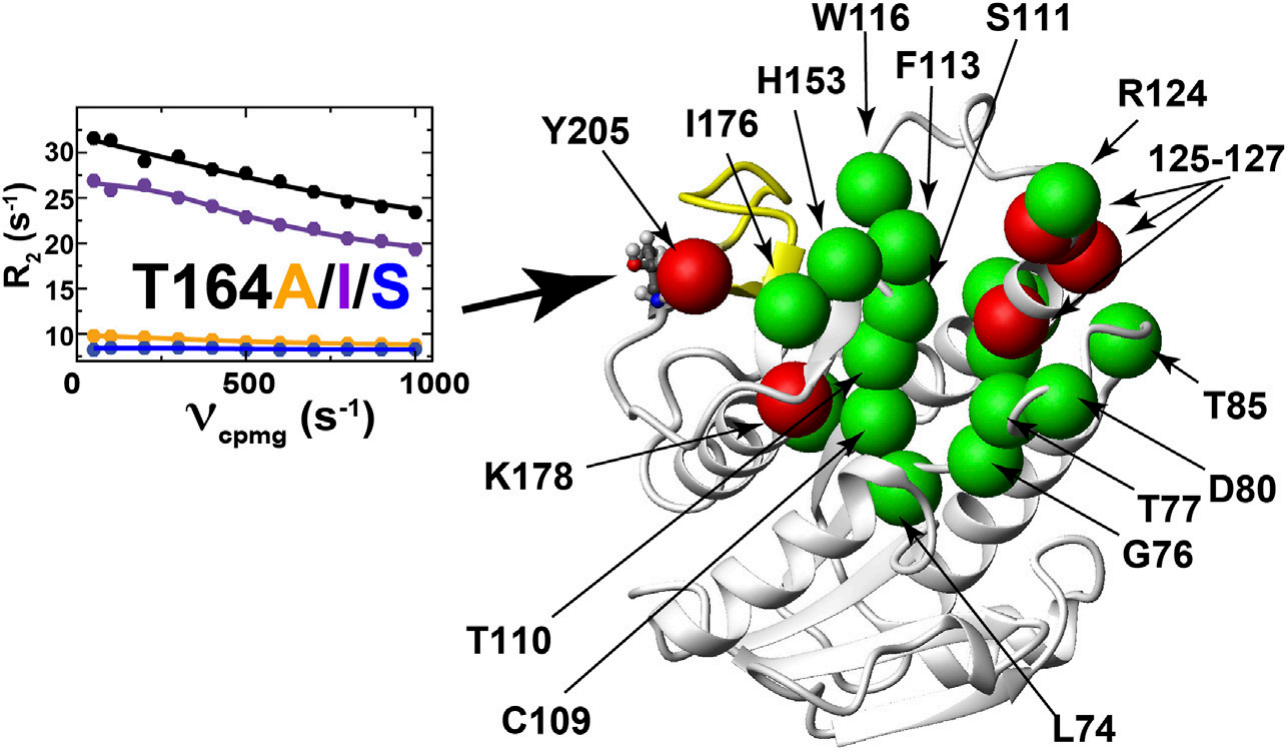
Mutations to BLVRB T164 induce long-range dynamic changes. R2-CPMG dispersion profiles for BLVRB WT (black), T164A (orange), T164I (purple), and T164S (blue) at position 164 are shown with the amide of distal changes mapped onto the X-ray crystal structure shown as either green spheres (R2-CPMG profiles that are differentially altered upon mutation to T164 along with S111 that is not differentially perturbed) or red spheres (R2-CPMG profiles that exhibit similar responses upon mutation to T164).

All three single site mutations induce changes to R2-CPMG dispersion profiles for residues that primarily surround the active site (Figure 7 and Supplementary Figure S2 for full R2 CPMG dispersions). Most interestingly, the T164S mutation quenches exchange at position 164 itself (Figure 7, left), which is exactly what is observed in lemur BLVRB that comprises an endogenous S164 (Figure 4D; Supplementary Figure S1). Overall, induced changes can broadly be separated into two groups. The first group includes largely localized changes with no clear patterns induced by each mutation (Figure 7, colored green and Supplementary Figure S2A that also illustrates several sites that do not change). For example, while active site residues such as T110, S111, F113, and H153 are adjacent to each other, each T164 mutation induces different changes to all of their R2-CPMG dispersion profiles. The second group includes several residues on both sides of the active site that exhibit similar patterns for each mutation (Figure 7, colored red and Supplementary Figure S2B). These include residues 125–127, H132, K178, and Y205 that exhibit similar profiles for both T164A and WT BLVRB but have higher amplitudes for T164I and lower amplitudes for T164S. While these amides of this second group are located on the periphery of the active site (Figure 7, red), it is important to note that their conformational dynamics could be coupled through side chain dynamics that include R124, F113, and H153. Thus, the RASSMM approach has identified imparted changes *via* position 164 that includes similar changes to this second group of residues.

Considering that both T164I and T164S mutants impact function with opposing effects to R2-CPMG dispersions, such findings reveal the localized nature (or segmental nature) of dynamics within this μs-ms timescale that has been shown to dominate dynamics in many systems that are still partially coupled (McDonald et al., 2012; Holliday et al., 2017). The similar perturbation patterns induced for the second group of R2-CPMG dispersions could theoretically be used as a rationale for globally fitting their R2-CPMG dispersions. Global fits of these residues or a subset of these residues of 125–127 indicate that this group moves slightly faster within both T164I and T164S relative to the WT BLVRB, T164A, and lemur BLVRB (Supplementary Table S4). It is plausible that faster motions may underlie the higher affinity of T164I and T164S for the coenzyme by driving sampling of binding competent conformations. However, further ensemble-based approaches to the panel of mutations produced here will likely be necessary to address the explicit mechanism by which these alterations to conformational sampling leads to higher coenzyme affinity. Finally, it is also important to note that a challenge in identifying networks in human BLVRB through changes to R2-CPMG dispersions is that several key residues surrounding the T164 site are simply not observed, which includes residues 167–175 and 199–204 (Figure 7, yellow ribbon). However, based on the imparted changes that can be measured, it is clear that while dynamics are coupled over large distances, they are also largely localized for many residues.

## Discussion

**FIGURE 8:**
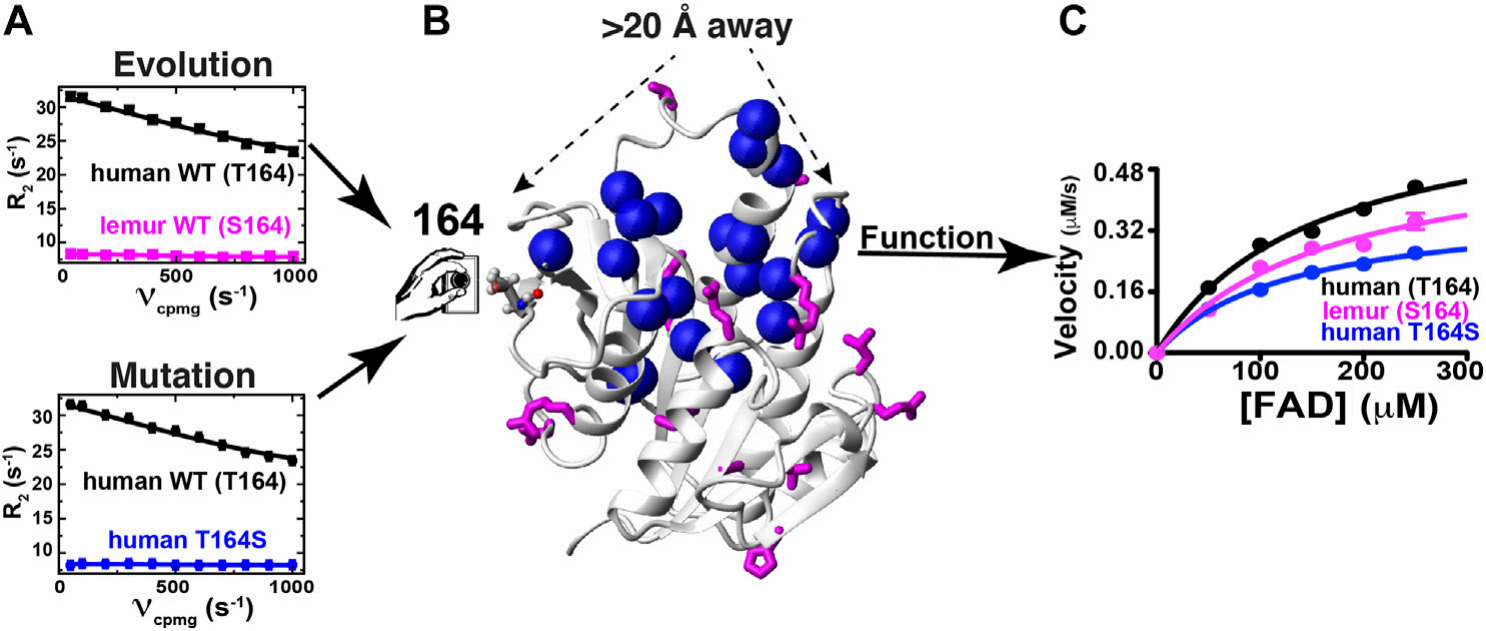
Evolution and mutagenesis “dial” dynamics and function in BLVRB. **(A)** R2-CPMG dispersion profiles are shown for human BLVRB WT (black) and lemur BLVRB WT (magenta) along with human BLVRB WT and human BLVRB T164S (blue). **(B)** Amides that exhibit changes to their R2-CPMG dispersion profiles (blue spheres) are shown together with residues differences between human and lemur BLVRB homologues (magenta). **(C)** Steady-state kinetics of human WT BLVRB, lemur WT BLVRB, and human BLVRB T164S.

The dynamic basis of enzyme catalysis is still an emerging field that has benefited from the advent of NMR relaxation studies such as those used here to begin identifying allosterically coupled networks within BLVRB. Allosteric regulation of enzyme function is increasingly recognized to occur through multiple mechanisms, which include mechanical coupling such as the classic case of hemoglobin but also dynamic coupling where motions influence networks of partially coupled movements. Unfortunately, the basis of long-range allosteric regulation is complicated owing to the nature of motions that are often segmental. For example, even after decades of studies on DHFR, studies are still revealing how global motions modulate this enzyme’s function (Boehr et al., 2006; Mauldin et al., 2012; Singh et al., 2015). Here, we have discovered that the evolutionarily changing position of 164 within BLVRBs also modulates function by a comparative analysis that includes multiple BLVRB homologues (i.e., human and lemur BLVRBs) and the application of the RASSMM approach that has identified both functional and dynamic changes. Strikingly, whether it is evolution or mutagenesis that positions a serine at position 164, the specific changes to dynamics at this site are identically quenched (Figure 8A). However, the segmental nature of motions identified within other enzymes is also evident in BLVRB (McDonald et al., 2012; Holliday et al., 2017), as monitored through the differential effects to R2-CPMG dispersions for different mutations to human BLVRB T164. None-the-less, networks of coupled motions such as those for human BLVRB T164S can be measured all the way to the active site (Figure 8B) and impart a 2-fold change to turnover (Figure 8C). Considering that mutations to active site residues such as BLVRB S111 impart approximately a 3-fold change to substrate turnover that dictates hematopoietic cell fate (Wu et al., 2016; Chu et al., 2017), the fact that catalysis can be modulated distally by a 2-fold change is significant. Our studies here therefore reveal that the dynamics of BLVRB can fine-tune function *via* dynamic allostery.

While the use here of multiple BLVRB homologues and multiple human BLVRB mutants illustrate how enzyme function may be “dialed” through allostery (Figure 8), there remain multiple questions that must be further addressed to understand the underlying mechanisms. For example, the dynamic effects imparted by human BLVRB T164 mutants are similar for residues on opposite sides of the active site and thus, side chain dynamics may be important to determine whether these serve to bridge dynamics. Most of these active site residues are aromatics, so the use of ^13^C-aromatic R2-CPMG experiments may be important to identify their differences (Weininger et al., 2012; Kasinath et al., 2013; Raum et al., 2018). Future studies that probe potential shifts to faster μs timescales using R1rho-type experiments may also reveal how these mutations allosterically modulate dynamics in the holo forms as well and how these may be related to faster timescale dynamics within the apo forms. Swapping evolutionarily changing sites identified *via* RASSMM such as those at position 164 within other homologues and monitoring their effects on dynamics and catalytic function will also be important to understand how such networks have evolved beyond human BLVRB. Finally, ensemble-based structural studies of these homologous and mutants may be especially informative to address the physical basis of allosteric regulation. While solution-based approaches would require an enormous amount of data, such as resonance assignments and NOEs for each mutant, newly developed X-ray crystallography approaches that directly visualize these ensembles may offer an alternative means (Keedy et al., 2018; Yabukarski et al., 2020).

In regard to BLVRB function, the human T164 mutations engineered here provides evidence that coenzyme-release is coupled to the rate-limiting step. Specifically, recent attempts to identify the rate-limiting step for BLVRB have been hampered by the complexity of ternary complex formation and a uniquely evolving active site. For example, coenzyme-release can be controlled *via* evolutionarily guided substitutions to the BLVRB active site, yet the FAD substrate appears to slow coenzyme-release in a manner that is dependent on these very same active site substitutions (Duff et al., 2020). In contrast, mutations to T164 here have allowed us to allosterically modulate function without directly perturbing the active site through. Specifically, as the coenzyme affinity becomes tighter for human BLVRB T164 mutations (Figure 5A), FAD substrate turnover is slowed (Figure 5B), which suggests that coenzyme binding is either the rate-limiting step or coupled to the rate-limiting step. Thus, we show here that allosterically modulating enzyme function may provide a general means for probing catalytic mechanism.

## Materials and Methods

### Protein Expression and Purification

All BLVRB proteins were purified as previously described (Paukovich et al., 2018; Duff et al., 2020). Briefly, all sequences encoding human BLVRB (NCBI Reference Sequence: NP_000704.1) and lemur BLVRB (NCBI Reference Sequence: XP_020138941.1) were codon optimized and synthesized as a Gibson block (Integrated DNA Technologies, Inc., ). For insertion into pET21b. All human BLVRB mutants were also constructed the same way. As human BLVRB T164S did not express in pET21b, it was recloned into pJ401k for expression. All constructs encoded an N-terminal 6xHis tag and thrombin cleavage site. Unlabeled proteins were grown in luria broth (LB) while labeled proteins were grown in M9 minimal media (6 g/L Na_2_HPO_4_, 3 g/L KH_2_PO_4_, 0.5 g/L NaCl, 1 g/L NH_4_Cl, 2 g/L glucose, 2 ml of 1 M MgSO_4_, 100 ml of 1 M NaCl CaCl_2_, 10 mg/L thiamine). ^15^N-labeled proteins were grown in M9 media with ^15^N ammonium chloride and ^2^H,^15^N-labeled proteins grown in 100% D_2_O with ^15^N ammonium chloride. Cell growths were induced with 0.1 mM isopropyl β-D-1-thiogalactopyranoside (IPTG). All proteins were purified in denaturing buffer (5 M guanidine, 50 mM Na_2_HPO_4_, pH 7.5, 500 mM NaCl, 10 mM imidazole) *via* Ni affinity (Sigma) and followed by application to a 3 ml Resource 15RPC (GE Healthcare Life Sciences) to remove any residual coenzyme prior to refolding through dialysis into 1 M arginine buffer (100 mM tris, pH 7.5, 100 mM NaCl, 1 mM DTT). Finally, proteins were dialyzed to NMR buffer (50 mM bis-tris, pH 6.5, 50 mM NaCl) and concentrated proteins were cleaved with thrombin (Sigma) to remove the 6x-His tag and applied to a superdex-75 (GE). Proteins were concentrated and frozen until further use.

### Thermodynamic and Kinetic Analysis

Isothermal titration calorimetry (ITC), steady-state, and pre-steady state experiments for BLVRB were performed identically to previous studies (Paukovich et al., 2018; Duff et al., 2020). Briefly, a MicroCal VP-ITC was used with samples containing 100 mM enzyme and NADP^+^ as the titrant at 1 mM with all buffers identical to that used for NMR (50 mM bis-tris, pH 6.5, 50 mM NaCl). ITC experiments were performed in duplicate at 20°C and processed using Origin software provided with the MicroCal VP-ITC. All reported values are the average and standard deviations of these duplicates. For UV-steady state kinetics that monitored initial velocities, a Biotek Synergy 2 multi-mode detection plate reader was used with 200 µl total volumes (pathlength of 0.625 cm), which monitored NADPH conversion to NADP^+^ at 340 nm, as previously described (Cunningham et al., 2000). Initial velocities were fit to the Michaelis-Menten equation using GraphPad Prism version 4.0 (GraphPad Software Inc, San Diego, CA). Velocities corrections to mM*s^−1^ were calculated using the extinction coefficient of NADPH of 6,222 M^−1^*cm^−1^. Pre-steady-state kinetics experiments were performed using an SX20 model Applied PhotoPhysics stopped-flow with 1 μs dead time. The pre-steady-state burst was fit to an exponential decay (the pre-steady-state rate of hydride transfer, k_hyd_) and a linear equation (the steady-state rate) using 3 FAD concentrations of 0.5, 1.0, and 1.5 mM using GraphPad Prism. As hydride transfer rates are concentration-independent (Fierke et al., 1987; Maglia et al., 2003), reported hydride transfer rates were averaged between these FAD concentrations with uncertainties calculated as their standard deviations.

### NMR Spectroscopy and Data Analysis

All BLVRB samples were prepared in 50 mM bis-tris, pH 6.5, 50 mM NaCl, 1 mM DTT at 500 mM enzyme with 5% D_2_O and data were collected on a Varian 900 spectrometer equipped with a cryo-probe at 20°C. Resonance assignments for the WT BLVRB previously published were used (Paukovich et al., 2018) and amide assignments for T164 mutants were confirmed *via* 3D^15^N-NOESY-HSQCs. Spectra were processed using NMRPipe (Delaglio et al., 1995) and all data were analyzed using CCPNmr software (Vranken et al., 2005). R2-CPMG dispersions were collected on ^2^H,^15^N-labeled proteins with TROSY selection as previously described (Schlegel et al., 2009; Holliday et al., 2017) and dispersions were fit to the full Carver-Richards equation using CPMG_fit as previously described (Schlegel et al., 2009). R1 relaxation experiments were collected on ^15^N-labeled proteins using the standard BioPack sequence with 0.01, 0.1, 0.3, 0.5 0.7, 0.9, and 1.1 s delays with R1 relaxation rates calculated in CCPNmr.

NMR Chemical shift projection analysis (CHESPA) was used to probe for potential covariance between human BLVRB T164 mutations and WT BLVRB coenzyme binding, as previously described (Selvaratnam et al., 2012a; Selvaratnam et al., 2012b; Gagne et al., 2015). Briefly, amide chemical shifts were exported from CCPNmr (Supplementary Table S2) and the cosine angles were calculated between CSPs induced for human WT BLVRB apo→holo previously published (BMRB accession 27,462 and 27,463) and each apo→T164 mutation described in this study (Supplementary Table S3).

### NMR Ensemble Calculations

Apo BLVRB ensembles were calculated with chemical shifts previously obtained (Paukovich et al., 2018) and supplemented with NOEs for structure determination using Resolution Adapted Structural RECombination (RASREC) in CS-Rosetta (Lange and Baker, 2012), as we have previously described (Holliday et al., 2015b). CCPNmr was used for spectral analysis with chemical shifts and NOEs exported for CS-Rosetta calculations. NOEs were derived from ^15^N-NOESY and ^13^C-NOESY spectra (Supplementary Table S1). Co-evolution restraints were also used as calculated from the GREMLIN server using the human BLVRB sequence (Kamisetty et al., 2013). Fragment libraries for CS-Rosetta were calculated without any homologues in order to avoid further biasing with final statistics of the 10 lowest energy structures shown in Table 1.

## Data Availability

The datasets presented in this study can be found in online repositories. The names of the repository/repositories and accession number(s) can be found in the article/Supplementary Material.

## Abbreviations

BLVRA, biliverdin reductase A; BLVRB, biliverdin reductase B; CPMG, Car-Purcell-Meiboom-Gill; NMR, nuclear magnetic resonance.
