# Supplementary material for: Modulating Enzyme Function via Dynamic Allostery within Biliverdin Reductase B
Source: Front Mol Biosci. 2021 May 20;8:691208. doi: 10.3389/fmolb.2021.691208 (PMC8173106; doi:10.3389/fmolb.2021.691208)
Supplement: Supplementary file 1 [file DataSheet1.docx]

**Supplementary Material**

**Redzic et al.**

**Modulating enzyme function via dynamic allostery within biliverdin reductase B.**

**Supplementary Figures.**


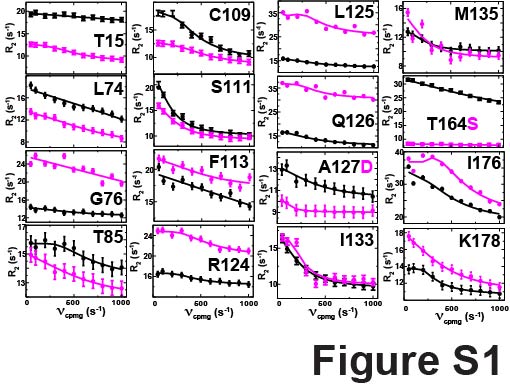


**Figure S1.** Comparison of dynamics between lemur and human BLVRB. R2-CPMG dispersion profiles are shown for lemur BLVRB (magenta) and human BLVRB (black) individually fit. All data were collected at 900 MHz at 20 ^o^C.

**___________________________**

**
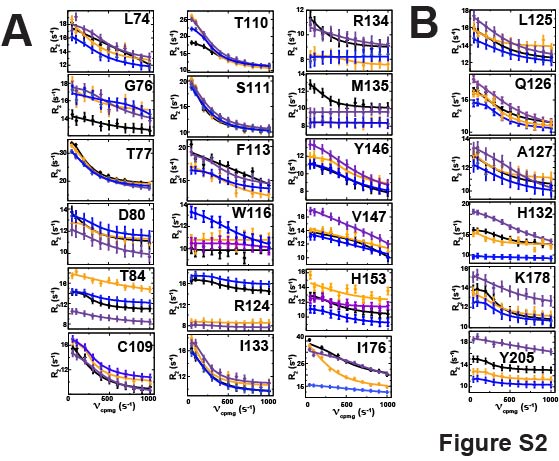
**

**Figure S2.** Comparison of dynamics between human BLVRB WT and T164 mutations. Individual fits are shown for each amide nitrogen for human WT BLVRB (black), T164A (orange), T164I (purple) and T164S (blue). A) R2-CPMG dispersion profiles altered upon T164 mutation with dissimilar changes for each mutant. B) R2-CPMG dispersion profiles altered upon T164 mutation with similar changes for each mutant. All data were collected at 900 MHz at 20 ^o^C.

**___________________________**

**Supplementary Tables.**

| **Residue 1** | **Residue type** | **Residue Atom** | **Residue 2** | **Residue type** | **Residue Atom** | **Upper Restraint (**Å**)** |
| --- | --- | --- | --- | --- | --- | --- |
| 175 | VAL | HA | 153 | HIS+ | H | 4.74 |
| 175 | VAL | HB | 153 | HIS+ | H | 3.97 |
| 107 | VAL | H | 73 | VAL | H | 4.31 |
| 106 | VAL | HA | 71 | VAL | H | 3.8 |
| 108 | ALA | HA | 73 | VAL | H | 4.14 |
| 144 | LEU | H | 142 | SER | HG | 3.85 |
| 27 | GLY | H | 25 | GLN | H | 5.2 |
| 182 | GLY | H | 180 | ASP- | H | 4.87 |
| 183 | HIS+ | H | 180 | ASP- | HA | 3.98 |
| 197 | HIS+ | H | 195 | ASP- | H | 3.16 |
| 197 | HIS+ | H | 195 | ASP- | H | 3.5 |
| 60 | ASP- | H | 57 | GLN | H | 4.45 |
| 61 | VAL | H | 59 | ALA | H | 4.28 |
| 70 | ALA | H | 68 | GLN | H | 4.4 |
| 25 | GLN | H | 23 | ALA | H | 4.69 |
| 19 | THR | H | 17 | LEU | H | 4.86 |
| 90 | GLY | H | 88 | SER | H | 3.99 |
| 100 | ALA | H | 97 | ALA | HA | 3.81 |
| 181 | LEU | H | 179 | HIS+ | H | 4.84 |
| 23 | ALA | H | 21 | ALA | H | 4.49 |
| 23 | ALA | H | 21 | ALA | H | 4.00 |
| 103 | VAL | H | 101 | HIS+ | H | 4.05 |
| 69 | ASP- | H | 5 | LYS+ | H | 4.39 |
| 60 | ASP- | H | 57 | GLN | H | 4.78 |
| 182 | GLY | H | 180 | ASP- | H | 4.92 |
| 96 | ALA | H | 92 | ARG+ | HA | 4.3 |
| 137 | LYS+ | H | 93 | ASN | H | 3.00 |
| 107 | VAL | H | 71 | VAL | H | 4.5 |
| 61 | VAL | H | 59 | ALA | H | 4.88 |
| 69 | ASP- | H | 6 | ILE | H | 5.15 |
| 69 | ASP- | H | 6 | ILE | HA | 4.47 |
| 69 | ASP- | H | 6 | ILE | QG2 | 4.45 |
| 70 | ALA | H | 6 | ILE | QG2 | 3.71 |
| 70 | ALA | H | 68 | GLN | HA | 3.85 |
| 146 | TYR | HA | 106 | VAL | H | 4.49 |
| 143 | GLY | H | 141 | GLU- | H | 4.29 |
| 143 | GLY | H | 141 | GLU- | H | 4.83 |
| 94 | ILE | H | 91 | ALA | HA | 3.89 |
| 139 | LEU | H | 137 | LYS+ | H | 4.18 |
| 95 | VAL | H | 92 | ARG+ | HA | 3.93 |
| 51 | VAL | H | 33 | LEU | HA | 4.11 |
| 36 | ASP- | H | 34 | VAL | HB | 4.22 |
| 22 | GLN | H | 19 | THR | HB | 4.26 |
| 19 | THR | H | 17 | LEU | H | 4.82 |
| 73 | VAL | HA | 9 | PHE | H | 4.37 |
| 25 | GLN | H | 23 | ALA | H | 4.63 |
| 191 | THR | H | 189 | LEU | H | 4.38 |
| 188 | CYS | H | 186 | LEU | H | 4.58 |
| 181 | LEU | H | 179 | HIS+ | H | 3.51 |
| 61 | VAL | H | 58 | ALA | HA | 3.85 |
| 128 | VAL | H | 125 | LEU | HA | 4.59 |
| 107 | VAL | H | 72 | ILE | HA | 4.33 |
| 98 | MET | H | 96 | ALA | H | 4.58 |
| 96 | ALA | H | 93 | ASN | HA | 4.3 |
| 64 | THR | H | 61 | VAL | HA | 3.91 |
| 93 | ASN | HD21 | 57 | GLN | H | 5.23 |
| 70 | ALA | H | 68 | GLN | H | 4.64 |
| 26 | ALA | H | 24 | VAL | H | 4.39 |
| 31 | THR | H | 7 | ALA | QB | 5.23 |
| 69 | ASP- | H | 6 | ILE | QG2 | 4.14 |
| 162 | ALA | H | 160 | THR | HB | 4.59 |
| 198 | SER | HA | 148 | ALA | QB | 3.69 |
| 177 | SER | HA | 155 | GLY | H | 3.99 |
| 62 | ASP- | H | 59 | ALA | HA | 4.06 |
| 64 | THR | HB | 61 | VAL | HA | 3.68 |
| 64 | THR | H | 61 | VAL | HA | 4.23 |
| 93 | ASN | HB3 | 58 | ALA | HA | 4.65 |
| 69 | ASP- | H | 3 | VAL | HA | 3.00 |
| 93 | ASN | HB2 | 58 | ALA | HA | 4.19 |
| 126 | GLN | H | 121 | VAL | QG1 | 5.62 |
| 162 | ALA | QB | 160 | THR | HB | 3.28 |
| 162 | ALA | QB | 160 | THR | HA | 4.02 |
| 192 | ASP- | HB3 | 156 | ASP- | HA | 3.00 |
| 198 | SER | HA | 148 | ALA | QB | 3.42 |
| 162 | ALA | QB | 160 | THR | HB | 3.48 |
| 188 | CYS | H | 186 | LEU | HA | 5.07 |
| 70 | ALA | H | 68 | GLN | HA | 5.34 |
| 61 | VAL | H | 58 | ALA | HA | 4.81 |
| 175 | VAL | HB | 153 | HIS+ | H | 4.49 |
| 22 | GLN | H | 19 | THR | HB | 5.19 |
| 108 | ALA | HA | 73 | VAL | H | 4.4 |
| 100 | ALA | H | 97 | ALA | HA | 3.00 |
| 106 | VAL | HA | 71 | VAL | H | 4.13 |
| 96 | ALA | H | 92 | ARG+ | HA | 4.68 |
| 36 | ASP- | H | 34 | VAL | HB | 4.68 |
| 175 | VAL | HA | 153 | HIS+ | H | 5.3 |
| 95 | VAL | H | 92 | ARG+ | HA | 5.1 |
| 93 | ASN | HB2 | 58 | ALA | HA | 4.23 |
| 70 | ALA | H | 6 | ILE | QG2 | 4.8 |
| 73 | VAL | HA | 9 | PHE | H | 3.84 |

**Table S1. Sparse NOEs used for human apo BLVRB CS-Rosetta ensemble calculations.**

Listed are the NOEs used for ensemble calculations extracted directly from CCPNmr greater than 2 residues apart.

**___________________________**

|  | WT Apo | | WT Holo | | T164S | | T164I | | T164A | |
| --- | --- | --- | --- | --- | --- | --- | --- | --- | --- | --- |
| **Res.** | **N** | **H** | **N** | **H** | **N** | **H** | **N** | **H** | **N** | **H** |
| 1 | 122.06775 | 8.36582 | 122.009 | 8.358 | 122.07513 | 8.3679 | 122.07904 | 8.3702 | 122.08272 | 8.3644 |
| 4 | 126.29739 | 9.18474 | 126.109 | 9.178 | 121.72742 | 8.3447 | 121.75373 | 8.35061 | 121.728 | 8.34265 |
| 5 | 122.84096 | 8.87995 | 122.84 | 8.859 | 126.31457 | 9.19001 | 126.34596 | 9.1941 | 126.29229 | 9.18338 |
| 6 | 115.83527 | 8.2541 | 115.74615 | 8.25703 | 122.87533 | 8.8846 | 122.85007 | 8.88532 | 122.87258 | 8.88107 |
| 7 | 120.83132 | 7.59258 | 120.859 | 7.682 | 115.9159 | 8.2559 | 115.89575 | 8.25883 | 115.92204 | 8.25485 |
| 8 | 117.13779 | 8.22314 | 116.989 | 8.303 | 120.83277 | 7.5904 | 120.84748 | 7.5981 | 120.85954 | 7.59572 |
| 9 | 127.41996 | 8.81426 | 127.047 | 8.91 | 117.15493 | 8.22323 | 117.13338 | 8.21887 | 117.15674 | 8.22285 |
| 10 | 114.69058 | 9.40038 | 115.869 | 9.944 | 127.4377 | 8.81669 | 127.44107 | 8.82122 | 127.42381 | 8.81624 |
| 11 | 121.89668 | 9.1829 | 120.465 | 8.94 | 114.70715 | 9.4038 | 114.71312 | 9.40496 | 114.69955 | 9.40062 |
| 12 | 97.68254 | 7.4815 | 95.70147 | 7.051 | 121.94778 | 9.1883 | 121.88834 | 9.18987 | 121.91692 | 9.18121 |
| 13 | 108.52903 | 7.35808 | 113.07 | 7.491 | 132.23545 | 7.4851 | 132.23272 | 7.48251 | 132.24151 | 7.47821 |
| 14 | 117.4519 | 8.65451 | 117.929 | 9.035 | 108.55035 | 7.36269 | 108.55617 | 7.36308 | 108.55235 | 7.35958 |
| 15 | 115.3007 | 8.47597 | 116.249 | 9.263 | 117.46013 | 8.65995 | 117.46127 | 8.65734 | 117.47275 | 8.65525 |
| 16 | 117.18005 | 8.819 | 118.873 | 9.664 | 115.31091 | 8.48081 | 115.29961 | 8.47839 | 115.30808 | 8.47916 |
| 17 | 121.39381 | 8.45998 | 120.442 | 8.344 | 117.19468 | 8.82337 | 117.17833 | 8.82588 | 117.18174 | 8.81869 |
| 18 | 111.88648 | 6.92026 | 110.9767 | 6.6488 | 121.42042 | 8.46654 | 121.42093 | 8.46553 | 121.40626 | 8.46492 |
| 19 | 123.35406 | 8.04543 | 123.855 | 8.117 | 111.88747 | 6.92341 | 111.86258 | 6.92005 | 111.87812 | 6.91892 |
| 20 | 121.54639 | 8.87445 | 121.597 | 9.171 | 123.36728 | 8.04609 | 123.28573 | 8.04628 | 123.34551 | 8.03877 |
| 21 | 120.12312 | 7.71521 | 119.886 | 7.54 | 121.58346 | 8.87898 | 121.51836 | 8.87787 | 121.58419 | 8.87567 |
| 22 | 116.06957 | 8.54351 | 115.976 | 8.518 | 120.12847 | 7.72063 | 120.14314 | 7.7227 | 120.13663 | 7.72035 |
| 23 | 124.11573 | 8.97418 | 124.099 | 9.026 | 116.07176 | 8.54262 | 116.0762 | 8.55482 | 116.07046 | 8.54105 |
| 24 | 119.08055 | 8.38822 | 118.917 | 8.385 | 124.13041 | 8.97909 | 124.13118 | 8.97696 | 124.1268 | 8.97508 |
| 25 | 120.60779 | 8.21417 | 120.544 | 8.198 | 119.09498 | 8.38991 | 119.10416 | 8.39378 | 119.08562 | 8.3896 |
| 26 | 119.14917 | 7.75045 | 119.103 | 7.737 | 120.62014 | 8.21609 | 120.62502 | 8.22007 | 120.62224 | 8.21569 |
| 27 | 104.51183 | 7.64948 | 104.387 | 7.638 | 119.16703 | 7.75531 | 119.16263 | 7.75713 | 119.16635 | 7.75366 |
| 28 | 117.94835 | 7.67474 | 117.821 | 7.632 | 104.52618 | 7.65242 | 104.53148 | 7.6546 | 104.52498 | 7.65126 |
| 29 | 125.81208 | 9.02241 | 125.692 | 8.994 | 117.96851 | 7.67852 | 117.96546 | 7.67988 | 117.97756 | 7.67761 |
| 30 | 131.25569 | 9.32488 | 131.278 | 9.358 | 125.83868 | 9.02426 | 125.84509 | 9.02596 | 125.85024 | 9.02537 |
| 31 | 125.73036 | 9.35125 | 125.961 | 9.39 | 131.26255 | 9.32746 | 131.28163 | 9.3278 | 131.28418 | 9.32433 |
| 32 | 115.71446 | 8.19281 | 115.593 | 8.249 | 125.74362 | 9.35037 | 125.73841 | 9.35626 | 125.74926 | 9.35352 |
| 33 | 122.18727 | 5.85007 | 121.462 | 5.91 | 115.73855 | 8.1971 | 115.73396 | 8.19621 | 115.7497 | 8.19085 |
| 34 | 121.7967 | 9.13367 | 123.522 | 9.449 | 122.2086 | 5.85346 | 122.22202 | 5.8558 | 122.21195 | 5.85315 |
| 35 | 122.577 | 8.73774 | 122.127 | 8.846 | 121.77856 | 9.13862 | 121.82262 | 9.13717 | 121.76546 | 9.13214 |
| 36 | 114.59069 | 7.43852 | 115.5884 | 7.83344 | 122.61802 | 8.74326 | 122.61951 | 8.74522 | 122.5938 | 8.74285 |
| 38 | 119.43731 | 8.79216 | 120.02 | 9.061 | 114.60362 | 7.43795 | 114.61226 | 7.4426 | 114.57789 | 7.43659 |
| 39 | 118.51527 | 7.4076 | 118.094 | 7.302 | 119.45507 | 8.79486 | 119.44782 | 8.79706 | 119.45213 | 8.79597 |
| 42 | 118.1159 | 8.77855 | 118.112 | 8.79 | 118.52744 | 7.40855 | 118.53787 | 7.41215 | 118.5313 | 7.40747 |
| 43 | 119.59167 | 7.94375 | 119.452 | 7.902 | 118.11845 | 8.78315 | 118.13056 | 8.78454 | 118.12036 | 8.78064 |
| 44 | 109.01466 | 8.20269 | 108.874 | 8.23 | 119.60379 | 7.94669 | 119.61321 | 7.94776 | 119.60105 | 7.94486 |
| 46 | 123.82517 | 8.85678 | 123.533 | 8.861 | 109.02718 | 8.20726 | 109.02632 | 8.20922 | 109.02109 | 8.20448 |
| 48 | 123.60035 | 7.63824 | 123.438 | 7.678 | 123.87166 | 8.86101 | 123.86313 | 8.86353 | 123.84861 | 8.85902 |
| 49 | 111.49007 | 7.97456 | 111.417 | 7.944 | 123.64891 | 7.64631 | 123.60317 | 7.64062 | 123.58633 | 7.63326 |
| 50 | 125.42049 | 8.96243 | 125.549 | 9.014 | 111.57625 | 7.97064 | 111.47641 | 7.98035 | 111.4363 | 7.97957 |
| 51 | 131.90937 | 9.05901 | 131.721 | 9.037 | 125.40926 | 8.95839 | 125.45549 | 8.97132 | 125.47088 | 8.9706 |
| 52 | 128.72026 | 8.45515 | 129.381 | 8.553 | 131.90795 | 9.05978 | 131.92559 | 9.0631 | 131.92966 | 9.05926 |
| 53 | 116.89778 | 8.6474 | 118.073 | 8.832 | 128.73482 | 8.4577 | 128.73185 | 8.46136 | 128.71733 | 8.45644 |
| 54 | 120.84066 | 9.57599 | 120.921 | 9.374 | 116.91367 | 8.65095 | 116.90146 | 8.65084 | 116.89129 | 8.64498 |
| 56 | 117.30571 | 8.24493 | 118.767 | 9.366 | 120.85448 | 9.58115 | 120.85557 | 9.58047 | 120.8473 | 9.5743 |
| 57 | 121.08479 | 8.14434 | 121.009 | 8.089 | 117.35445 | 8.25056 | 117.34667 | 8.25111 | 117.31925 | 8.24754 |
| 58 | 130.84825 | 9.0777 | 130.897 | 9.107 | 121.09884 | 8.14835 | 121.09397 | 8.14766 | 121.09042 | 8.14422 |
| 59 | 118.02613 | 9.00449 | 118.007 | 9.027 | 130.87569 | 9.08057 | 130.86489 | 9.08359 | 130.86128 | 9.08162 |
| 60 | 119.66542 | 7.12145 | 119.633 | 7.183 | 118.02479 | 9.0062 | 118.05539 | 9.01146 | 118.05141 | 9.00798 |
| 61 | 121.65933 | 7.57684 | 121.739 | 7.603 | 119.65547 | 7.12614 | 119.67816 | 7.12482 | 119.67424 | 7.11925 |
| 62 | 121.93176 | 8.76552 | 121.96 | 8.807 | 121.72878 | 7.58107 | 121.68745 | 7.5831 | 121.67889 | 7.58221 |
| 63 | 113.457 | 7.24138 | 113.324 | 7.283 | 121.90516 | 8.76518 | 121.94961 | 8.77193 | 121.83515 | 8.76445 |
| 64 | 116.2451 | 7.39827 | 116.069 | 7.407 | 113.46679 | 7.24461 | 113.49417 | 7.24672 | 113.46815 | 7.24395 |
| 65 | 118.28461 | 7.79852 | 118.022 | 7.75 | 116.24823 | 7.40362 | 116.25948 | 7.40283 | 116.25851 | 7.39828 |
| 67 | 109.83991 | 8.25479 | 109.795 | 8.237 | 118.34148 | 7.80408 | 118.29481 | 7.80218 | 118.25379 | 7.79701 |
| 68 | 115.11376 | 7.36579 | 114.969 | 7.326 | 109.83729 | 8.24488 | 109.86052 | 8.26195 | 109.85663 | 8.26524 |
| 69 | 116.15972 | 9.19246 | 116.06 | 9.193 | 115.13467 | 7.36895 | 115.11844 | 7.36981 | 115.11319 | 7.36449 |
| 70 | 117.18355 | 7.84663 | 117.023 | 7.813 | 116.15911 | 9.19447 | 116.16446 | 9.19682 | 116.17755 | 9.19676 |
| 71 | 116.93539 | 8.32312 | 117.37628 | 8.3174 | 117.20202 | 7.85138 | 117.20224 | 7.85094 | 117.19566 | 7.8474 |
| 72 | 125.31683 | 9.08657 | 125.651 | 9.106 | 116.93095 | 8.32492 | 116.94139 | 8.32715 | 116.96739 | 8.32533 |
| 73 | 127.34417 | 9.22649 | 127.253 | 9.129 | 125.33817 | 9.08969 | 125.33123 | 9.09016 | 125.32395 | 9.08982 |
| 74 | 132.25551 | 8.64281 | 132.538 | 8.634 | 127.36843 | 9.22791 | 127.37572 | 9.23662 | 127.35422 | 9.22822 |
| 76 | 109.01908 | 8.46757 | 107.199 | 7.111 | 132.25333 | 8.6482 | 132.26818 | 8.64679 | 132.25105 | 8.64673 |
| 77 | 111.66664 | 8.26481 | 107.909 | 8.495 | 109.04963 | None | 109.03702 | 8.46908 | 109.03029 | 8.46615 |
| 80 | 120.51793 | 8.01837 | 120.799 | 8.057 | 111.70172 | 8.27018 | 111.70051 | 8.27222 | 111.70541 | 8.26514 |
| 81 | 122.39928 | 8.4524 | 129.46 | 8.968 | 117.86618 | 8.534 | 117.81411 | 8.53009 | 117.85893 | 8.52887 |
| 82 | 117.83316 | 8.06128 | 119.36 | 8.353 | 120.50898 | 8.01973 | 120.51202 | 8.02278 | 120.4963 | 8.01619 |
| 85 | 112.70568 | 8.02091 | 112.855 | 8.047 | 117.85477 | 8.06663 | 117.85643 | 8.06621 | 117.84855 | 8.06361 |
| 86 | 118.65673 | 8.07885 | 121.873 | 8.477 | 112.72305 | 8.02282 | 112.71289 | 8.02338 | 112.69266 | 8.01759 |
| 88 | 117.18049 | 8.98268 | 117.359 | 8.809 | 118.68768 | 8.09567 | 118.70123 | 8.09182 | 118.67244 | 8.10174 |
| 89 | 123.74445 | 8.79886 | 124.149 | 9.002 | 117.18431 | 8.98443 | 117.21104 | 8.98864 | 117.18056 | 8.9826 |
| 90 | 108.22448 | 8.12752 | 108.144 | 8.068 | 123.78633 | 8.79478 | 123.77771 | 8.79806 | 123.68588 | 8.7646 |
| 92 | 118.65918 | 8.15413 | 119.028 | 8.158 | 108.23509 | 8.134 | 108.22726 | 8.13404 | 108.21609 | 8.1329 |
| 93 | 118.26912 | 7.6514 | 118.34 | 7.681 | 118.66822 | 8.15631 | 118.67169 | 8.15989 | 118.65531 | 8.15571 |
| 94 | 124.04394 | 8.41061 | 124.329 | 8.559 | 118.28278 | 7.65335 | 118.27935 | 7.659 | 118.27317 | 7.65672 |
| 95 | 119.2496 | 8.67596 | 119.422 | 8.768 | 124.04822 | 8.41458 | 124.0592 | 8.41712 | 124.06974 | 8.4141 |
| 96 | 120.50696 | 7.63839 | 120.467 | 7.591 | 119.27107 | 8.67784 | 119.23724 | 8.68092 | 119.27167 | 8.67686 |
| 97 | 120.49281 | 7.79777 | 120.458 | 7.805 | 120.51102 | 7.63915 | 120.51542 | 7.64477 | 120.50454 | 7.64227 |
| 98 | 119.37048 | 8.93212 | 119.552 | 8.983 | 120.53546 | 7.80288 | 120.50794 | 7.80285 | 120.48874 | 7.79929 |
| 99 | 117.5222 | 8.39537 | 117.571 | 8.425 | 119.35713 | 8.93802 | 119.40068 | 8.93996 | 119.40389 | 8.93491 |
| 100 | 120.97104 | 7.83498 | 120.795 | 7.836 | 117.51625 | 8.39469 | 117.57289 | 8.40224 | 117.59324 | 8.40372 |
| 101 | 111.97279 | 7.65231 | 111.72 | 7.633 | 121.067 | 7.8351 | 120.96875 | 7.84132 | 120.90332 | 7.84016 |
| 102 | 110.99857 | 7.67032 | 110.987 | 7.667 | 112.12709 | 7.66686 | 111.95797 | 7.65516 | 111.85839 | 7.64359 |
| 103 | 122.39928 | 8.4524 | 122.353 | 8.465 | 111.01024 | 7.67512 | 111.01273 | 7.67486 | 111.01191 | 7.67043 |
| 104 | 125.23735 | 8.84884 | 125.237 | 8.876 | 122.4285 | 8.45809 | 122.40754 | 8.45615 | 122.39811 | 8.45065 |
| 105 | 118.93898 | 6.63436 | 118.848 | 6.621 | 125.25846 | 8.85313 | 125.26629 | 8.85653 | 125.26273 | 8.85527 |
| 106 | 126.30872 | 9.3271 | 126.246 | 9.316 | 118.96275 | 6.63717 | 118.97089 | 6.63748 | 118.96992 | 6.63484 |
| 107 | 129.0105 | 8.63281 | 129.254 | 8.7 | 126.32174 | 9.33218 | 126.32726 | 9.33632 | 126.32033 | 9.33309 |
| 108 | 125.32953 | 8.66296 | 125.183 | 8.613 | 129.03434 | 8.63668 | 129.03034 | 8.63634 | 129.04702 | 8.63594 |
| 109 | 124.85179 | 8.21764 | 126.068 | 8.358 | 125.33675 | 8.66522 | 125.36092 | 8.66679 | 125.34442 | 8.66616 |
| 111 | 115.0714 | 8.0553 | 113.332 | 7.95 | 119.35713 | 8.93802 | 119.40068 | 8.93996 | 119.40389 | 8.93491 |
| 113 | 115.51694 | 7.93173 | 114.32684 | 7.72648 | 115.0898 | 8.06738 | 115.09496 | 8.07088 | 115.09998 | 8.07431 |
| 114 | 121.65933 | 7.57684 | 121.76424 | 7.60334 | 115.69191 | 7.96561 | 115.5348 | 7.94888 | 115.64983 | 7.96718 |
| 116 | 121.43909 | 7.44261 | 121.382 | 7.403 | 121.72878 | 7.58107 | 121.68745 | 7.5831 | 121.67889 | 7.58221 |
| 119 | 108.42145 | 8.47727 | 108.54203 | 8.50419 | 121.50085 | 7.4584 | 121.49264 | 7.46317 | 121.50701 | 7.46579 |
| 120 | 120.60779 | 8.21417 | 120.544 | 8.198 | 108.32876 | 8.46982 | 108.34159 | 8.4747 | 108.25082 | 8.46185 |
| 121 | 122.83347 | 7.16075 | 122.612 | 7.084 | 122.85542 | 7.14846 | 122.85647 | 7.15546 | 122.83542 | 7.13761 |
| 124 | 115.60898 | 8.66425 | 115.523 | 8.729 | 117.52368 | 7.90906 | 117.51406 | 7.91587 | 115.55561 | 8.6755 |
| 125 | 117.49045 | 7.91983 | 117.00658 | 7.8365 | 124.94366 | 7.66093 | 124.94235 | 7.66293 | 117.50943 | 7.90183 |
| 126 | 124.95026 | 7.65995 | 125.229 | 7.714 | 122.43494 | 8.54985 | 122.44554 | 8.55133 | 124.91417 | 7.65556 |
| 127 | 122.39938 | 8.54701 | 123.014 | 8.49 | 118.36818 | 8.31183 | 118.37808 | 8.31351 | 122.44708 | 8.54673 |
| 128 | 118.3664 | 8.30832 | 119.32 | 8.422 | 121.92386 | 8.91979 | 121.90684 | 8.90181 | 118.36058 | 8.30948 |
| 130 | 121.90479 | 8.91927 | 121.837 | 9.093 | 119.92987 | 7.40102 | 119.93299 | 7.40242 | 121.89772 | 8.90564 |
| 131 | 119.90803 | 7.39823 | 120.145 | 7.344 | 121.15224 | 9.0486 | 121.16486 | 9.04586 | 119.91969 | 7.3981 |
| 133 | 121.11811 | 9.038 | 120.645 | 9.044 | 119.16703 | 7.75531 | 119.16263 | 7.75713 | 121.17103 | 9.0499 |
| 134 | 119.14917 | 7.75045 | 119.18 | 7.747 | 121.10527 | 8.88791 | 121.10619 | 8.89259 | 119.16635 | 7.75366 |
| 135 | 121.11798 | 8.89586 | 121.235 | 8.953 | 119.9148 | 9.01747 | 119.88097 | 9.01573 | 121.0211 | 8.87819 |
| 136 | 119.87456 | 9.0127 | 119.75231 | 8.97386 | 118.66822 | 8.15631 | 118.67169 | 8.15989 | 119.87202 | 9.01249 |
| 137 | 118.65918 | 8.15413 | 118.508 | 8.036 | 117.86027 | 7.53968 | 117.84672 | 7.54146 | 118.65531 | 8.15571 |
| 138 | 117.82161 | 7.5365 | 117.742 | 7.517 | 119.05075 | 7.67259 | 119.03176 | 7.67239 | 117.8409 | 7.5381 |
| 139 | 119.00796 | 7.667 | 118.831 | 7.673 | 124.13289 | 9.17404 | 124.1317 | 9.17745 | 119.03035 | 7.66877 |
| 140 | 124.12132 | 9.17027 | 124.217 | 9.191 | 116.09578 | 8.05313 | 116.09733 | 8.05588 | 124.11962 | 9.171 |
| 141 | 116.08512 | 8.05067 | 116.036 | 7.993 | 113.46679 | 7.24461 | 113.49417 | 7.24672 | 116.09955 | 8.05344 |
| 142 | 113.457 | 7.24138 | 113.423 | 7.241 | 110.42439 | 7.72497 | 110.42316 | 7.72425 | 113.46815 | 7.24395 |
| 143 | 110.40929 | 7.72219 | 110.328 | 7.706 | 131.95275 | 8.75538 | 131.97014 | 8.75677 | 110.42509 | 7.7233 |
| 146 | 126.81658 | 8.16983 | 126.896 | 8.164 | 130.22609 | 7.22915 | 130.24745 | 7.22202 | 126.80451 | 8.17706 |
| 147 | 131.94873 | 8.75833 | 131.92 | 8.76 | 127.84948 | 8.47746 | 127.78725 | 8.47888 | 131.95768 | 8.74885 |
| 148 | 130.22035 | 7.22574 | 130.307 | 7.242 | 129.1978 | 8.94796 | 129.26997 | 8.956 | 130.2177 | 7.21742 |
| 149 | 127.76914 | 8.46639 | 128.005 | 8.358 | 116.88141 | 7.26287 | 116.77573 | 7.27782 | 127.79566 | 8.47619 |
| 150 | 129.19484 | 8.94032 | 129.171 | 8.94 | 126.18057 | 7.49565 | 126.23103 | 7.51623 | 129.21912 | 8.95744 |
| 153 | 116.83949 | 7.27052 | 114.58359 | 7.27794 | 118.14983 | 8.23701 | 118.13196 | 8.24141 | 116.69997 | 7.28857 |
| 154 | 126.20659 | 7.50217 | 123.47276 | 7.53476 | 121.92386 | 8.91979 | 121.90684 | 8.90181 | 126.28868 | 7.5175 |
| 156 | 118.12904 | 8.23179 | 117.932 | 8.047 | 119.56177 | 7.86347 | 119.64328 | 7.87147 | 118.16706 | 8.23643 |
| 157 | 121.90479 | 8.91927 | 121.51477 | 8.74918 | 119.49324 | 10.21559 | 119.44093 | 10.18237 | 121.89772 | 8.90564 |
| 159 | 119.57469 | 7.85976 | 119.481 | 7.847 | 112.56227 | 9.06643 | 112.67845 | 9.12548 | 119.56939 | 7.8624 |
| 161 | 112.5886 | 9.07539 | 112.532 | 9.049 | 122.91642 | 8.03138 | 123.0065 | 8.04927 | 112.58611 | 9.07356 |
| 162 | 122.8983 | 8.00591 | 122.83 | 8.013 | 113.59874 | 5.88324 | 113.40538 | 5.88802 | 122.96337 | 8.01045 |
| 163 | 113.31982 | 5.86088 | 113.321 | 5.861 | 125.25846 | 8.85313 | 125.26629 | 8.85653 | 113.87621 | 5.87844 |
| 165 | 125.23735 | 8.84884 | 125.528 | 8.841 | 125.67456 | 8.33093 | 125.68212 | 8.33256 | 125.26273 | 8.85527 |
| 169 | 102.87822 | 7.32411 | 102.95432 | 7.31252 | 102.76136 | 7.35179 | 102.81478 | 7.35916 | 102.73435 | 7.36814 |
| 176 | 121.51705 | 8.63283 | 121.5393 | 8.57879 | 121.94778 | 9.1883 | 121.36289 | 8.60423 | 121.60295 | 8.61296 |
| 177 | 121.89668 | 9.1829 | 121.648 | 9.205 | 124.66148 | 9.09152 | 121.88834 | 9.18987 | 121.91692 | 9.18121 |
| 179 | 117.71027 | 7.21706 | 117.562 | 7.248 | 117.72317 | 7.2165 | 117.71463 | 7.20268 | 117.71834 | 7.20689 |
| 180 | 120.94356 | 7.25677 | 120.697 | 7.167 | 121.01846 | 7.25763 | 121.02394 | 7.28258 | 121.04455 | 7.26572 |
| 181 | 121.59147 | 8.74381 | 121.535 | 8.734 | 121.77817 | 8.75901 | 121.56566 | 8.73916 | 121.54309 | 8.72383 |
| 182 | 106.42732 | 8.10974 | 106.474 | 8.12 | 106.45459 | 8.1088 | 106.46555 | 8.11816 | 106.44504 | 8.10655 |
| 183 | 122.04261 | 8.03709 | 121.859 | 8.031 | 122.07532 | 8.0404 | 122.08037 | 8.04031 | 122.07281 | 8.04134 |
| 184 | 121.59147 | 8.74381 | 121.627 | 8.717 | 121.77817 | 8.75901 | 121.56566 | 8.73916 | 121.54309 | 8.72383 |
| 185 | 118.27944 | 8.43554 | 118.116 | 8.453 | 118.28648 | 8.44525 | 118.3284 | 8.44096 | 118.27626 | 8.44015 |
| 186 | 113.18401 | 7.38447 | 113.095 | 7.396 | 113.20278 | 7.37935 | 113.23143 | 7.39834 | 113.20493 | 7.37449 |
| 187 | 125.09218 | 8.17725 | 125.549 | 8.284 | 125.13022 | 8.18765 | 125.06802 | 8.1625 | 125.16333 | 8.19051 |
| 188 | 116.43553 | 7.58818 | 116.368 | 7.571 | 116.46796 | 7.59689 | 116.44962 | 7.58511 | 116.46437 | 7.59755 |
| 189 | 114.09819 | 7.57742 | 114.1 | 7.618 | 114.09292 | 7.58122 | 114.10221 | 7.57959 | 114.0967 | 7.58017 |
| 190 | 106.53178 | 7.72115 | 106.399 | 7.726 | 106.54841 | 7.72215 | 106.53368 | 7.7273 | 106.5461 | 7.72088 |
| 191 | 114.84025 | 7.19476 | 114.782 | 7.191 | 114.85654 | 7.19723 | 114.86923 | 7.20424 | 114.86881 | 7.19763 |
| 192 | 125.21727 | 8.31286 | 125.265 | 8.314 | 125.22758 | 8.31497 | 125.23508 | 8.31962 | 125.2105 | 8.31423 |
| 193 | 122.06775 | 8.36582 | 122.009 | 8.358 | 122.07513 | 8.3679 | 122.07904 | 8.3702 | 122.08272 | 8.3644 |
| 195 | 123.08123 | 7.45169 | 123.121 | 7.45 | 123.04695 | 7.45382 | 123.06311 | 7.45736 | 123.0508 | 7.45239 |
| 196 | 111.09279 | 8.67516 | 111.104 | 8.637 | 111.05947 | 8.67952 | 111.0575 | 8.68439 | 111.0472 | 8.68546 |
| 197 | 116.35555 | 8.31277 | 116.325 | 8.319 | 116.36473 | 8.31881 | 116.32421 | 8.30756 | 116.32263 | 8.3177 |
| 198 | 116.76308 | 8.85048 | 116.679 | 8.817 | 116.65161 | 8.88111 | 116.73275 | 8.8677 | 116.63303 | 8.88131 |
| 205 | 123.591 | 8.38656 | 123.704 | 8.392 | 123.53852 | 8.40426 | 123.35293 | 8.39812 | 123.46623 | 8.42493 |
| 206 | 125.95508 | 8.16707 | 125.825 | 8.147 | 125.94929 | 8.16586 | 125.95161 | 8.1513 | 125.97039 | 8.1588 |

**Table S2. Amide chemical shifts used for covariance analysis.**

Shifts represent direct output per residue (Res.) from CCPNmr for human BLVRB WT apo, WT holo, and the apo of T164S, T164I, and T164A.

**___________________________**

| **Residue** | **T164S** | **T164I** | **T164A** |
| --- | --- | --- | --- |
| 90 |  |  | -0.998 |
| 114 | -0.991 |  | -1.000 |
| 119 |  |  | -0.980 |
| 120 |  |  | -0.888 |
| 135 |  |  | 0.904 |
| 153 |  |  | -0.848 |
| 159 |  | 0.809 |  |
| 161 | 0.965 | 0.998 |  |
| 162 | 0.652 | 0.084 |  |
| 169 | 0.976 | 0.840 | 0.942 |
| 176 |  | -0.616 | -0.808 |
| 180 |  | 0.999 |  |
| 181 | 0.946 |  |  |
| 184 | 0.127 |  |  |
| 190 | 0.458 |  |  |
| 198 |  |  | 0.394 |
| 205 |  | 0.890 | 0.334 |

**Table S3. Chemical shift projection analysis for human BLVRB T164 mutants.**

Shown are the cosine angles between the basis vector produced from apo🡪holo WT BLVRB compared to the WT🡪T164 mutation listed using amide chemical shifts listed in Table S3 and computed using CHESPA, as described in Materials and Methods.

| **BLVRB** | **164 coupled^a^**  **kex (s^-1^)** | **Residues 125-127b**  **kex (s^-1^)** |
| --- | --- | --- |
| human WT (T164) | 2020±230 | 1960±300 |
| Human T164A | 1410±300 | 1160±300 |
| Human T164I | 3300±350 | 3800±800 |
| Human T164S | 2430±230 | 2360±360 |
| Lemur WT | 1500±400 | 1340±600 |

**Table S4. Exchange rates derived from global fits of R2-CPMG dispersions.**

a) Residues that exhibit similar changes upon mutation of human BLVRB T164 shown in Figure S2B were simultaneously fit. These include 125-127, H132, K178, and Y205. The amide of H132 was not observed within lemur WT BLVRB.

b) Residues 125-127 were simultaneously fit.

**___________________________**
